# Role of nuclear receptors, lipid metabolism, and mitochondrial function in the pathogenesis of diabetic kidney disease

**DOI:** 10.1152/ajprenal.00110.2025

**Published:** 2025-08-19

**Authors:** Eleni Hughes, Xiaoxin X. Wang, Lily Sabol, Keely Barton, Sujit Hegde, Komuraiah Myakala, Ewa Krawczyk, Avi Rosenberg, Moshe Levi

**Affiliations:** 1 Department of Biochemistry and Molecular & Cellular Biology, Georgetown University Medical Center, Washington, District of Columbia, United States;; 2 Department of Pathology, Center for Cell Reprogramming, Georgetown University Medical Center, Washington, District of Columbia, United States;; 3 Renal Pathology Service, Department of Pathology, Johns Hopkins University School of Medicine, Baltimore, Maryland, United States

**Keywords:** diabetic kidney disease, inflammation, lipids, mitochondria, nuclear receptors

## Abstract

Diabetic kidney disease (DKD) is a leading cause of end-stage renal disease (ESRD) and remains a significant clinical challenge due to its complex pathogenesis. This review explores the intricate interplay of metabolic, inflammatory, and cellular mechanisms that drive DKD progression, with a particular focus on lipid metabolism, mitochondrial dysfunction, oxidative stress, inflammation, cell injury, and epigenetic modifications. Advances in histopathological and molecular studies have expanded our understanding of glomerular, tubular, and vascular abnormalities in DKD, highlighting the critical role of nuclear hormone receptors, transcription factors, and G protein-coupled receptors in regulating renal lipid accumulation, mitochondrial function, inflammation, oxidative stress, and fibrotic pathways. In addition, emerging evidence implicates novel cell death mechanisms, including ferroptosis, necroptosis, pyroptosis, and PANoptosis, in DKD pathology. Epigenetic modifications, including DNA methylation, histone modifications, and noncoding RNAs, further contribute to disease progression by regulating gene expression in response to metabolic stress. As current therapeutic strategies remain insufficient to prevent DKD progression, this review also discusses novel molecular targets and emerging therapeutic approaches aimed at mitigating lipid toxicity, enhancing mitochondrial function, and suppressing inflammation. By integrating insights from histopathology, molecular biology, and translational research, this review provides a comprehensive framework for developing future strategies to delay or prevent DKD progression.

## INTRODUCTION

Diabetic kidney disease (DKD) is a major complication of diabetes, accounting for nearly half of new end-stage renal disease (ESRD) cases globally (1). DKD is characterized by a progressive decline in renal function, which manifests as albuminuria, reduced glomerular filtration rate, and histological abnormalities such as glomerulosclerosis and tubulointerstitial fibrosis. The pathogenesis of DKD is complex, involving the interplay of hemodynamic, metabolic, and inflammatory factors (2–5). Although traditional therapeutic approaches have targeted hyperglycemia and hypertension, many patients continue to experience disease progression, underscoring the need for novel insights into the molecular mechanisms underpinning DKD. This review will examine the role of nuclear hormone receptors, lipid metabolism, and mitochondrial function in the pathogenesis of inflammation, oxidative stress, cell injury, and fibrosis, all hallmarks of diabetic kidney disease.

## SPECTRUM OF HISTOPATHOLOGY

The link between diabetes and kidney disease was first noted in the 19th century through clinical observations of proteinuria in patients with diabetes. In the 20th century, Kimmelstiel and Wilson (6) described nodular glomerulosclerosis/intercapillary glomerulosclerosis (7), now recognized as the hallmark lesion of diabetic nephropathy (DN). With larger biopsy cohorts, advances in bright-field microscopy and electron microscopy, intermediate phases of glomerular injury, including glomerular and tubular basement membrane thickening (8–14), podocyte injury (15), and earlier mesangiopathic changes were described. The resulting functional disruptions, such as charge loss in the glomerular basement membrane (GBM) (16), and structural pores/tunnels (17) affecting charge and size selectivity, were also identified. The histopathologic spectrum of DN now includes abnormalities in all renal parenchymal compartments: glomerular, tubulointerstitial, and vascular. Initially, mesangial cell involvement was emphasized, later expanding to endothelial and podocyte dysregulation, most recently evolving to a more holistic understanding of cellular aberrations, especially with the advent of single-cell technologies. Every kidney compartment exhibits resident and reactive inflammatory cell alterations in DN, necessitating a focus on injury patterns and pathomechanistic insights into the underlying molecular and metabolic dysregulation.

### Glomerular Pathology

Few studies have documented DN’s natural history on serial biopsies, but those conducted show that glomerular phenotypes (glomerular volume, foot process effacement, GBM thickening) and arteriolar hyalinosis correlate with systemic metabolic dysregulation and antihypertensive treatment (18–20). Cellular aberrations involve mesangial cells, podocytes, endothelial cells, and inflammatory infiltrates. GBM thickening is initially detectable via electron microscopy but later becomes apparent under bright-field microscopy, especially on Periodic Acid–Schiff stain. Progressive mesangial matrix expansion may lead to acellular nodules with matrix and hyaline insudation (21). Capillary loop remodeling includes mesangiolysis (22), microaneurysmal dilatation, and endotheliosis (23). Microaneurysms are strongly associated with DN progression and Kimmelstiel-Wilson nodule (24). Hyalinosis can affect any endothelial-associated compartment, including Bowman’s capsule (capsular drop) and arterioles (25, 26). Segmental sclerosis, particularly “tip-lesions” at the glomerulotubular junction, is linked to hyperfiltration injury (27), and increased incidence of atubular glomeruli have been noted (28).

### Cellular Alterations in the Glomerulus

- Mesangial cells: exposure to high glucose increases collagen IV, fibronectin, and laminin expression (9, 29). Intraglomerular transforming growth factor-beta (TGF-β) activation in response to hyperglycemia (30) and advanced glycation end-products (AGEs) in mesangium (31) drive matrix expansion via TGF-β and inflammatory pathways.
- Endothelial cells: hyperglycemia induces endothelialto-mesenchymal transition (EndMT), promoting capillary rarefaction and fibrosis. Podocyte detachment and reduced endothelial fenestration correlate with macroalbuminuria and DN progression (32).
- Podocytes: foot process width correlates with proteinuria (15, 33). Oxidative stress and cytoskeletal disruption contribute to podocyte injury, leading to podocyturia and nephrinuria (34), a process that rapidly occurring in experimental models but is preventable with antioxidants (35). Urinary nephrin correlates with albuminuria, glomerulomegaly, and progressive nephropathy (36). GBM glycation impairs focal adhesion kinase and mitogen-activated protein kinase (MAPK) activity (37), whereas podocyte autophagy protects against albuminuria in diabetic endothelial injury (38).
- Parietal epithelial cells: in rodent models and human DN, parietal epithelial cells exhibit vacuolization and hypertrophy (39).

### Tubular Injury

The Armanni–Ebstein lesion, characterized by tubular epithelial vacuolation at the corticomedullary junction, is seen in diabetic ketoacidosis (40, 41). Excessive glucose uptake leads to mitochondrial dysfunction, inflammation, and tubular injury. Even in early DN, tubular epithelial alterations, nuclear changes, and mitochondrial injury occur (42). Tubular epithelial cells contribute to inflammation and fibrosis, generating collagen III and IV (43). Osteopontin overexpression in diabetic mice promotes macrophage infiltration and fibrosis, which is reduced upon knockout (44). TGF-β signaling is crucial in tubulointerstitial inflammation, with myostatin-induced ROS release upregulating NADPH oxidase and interstitial fibrosis (45). Yes-associated protein (YAP) expression in proximal tubules triggers connective tissue growth factor (CTGF), converting fibroblasts to myofibroblasts (46). Farnesoid X receptor (FXR) downregulation in tubular cells via TGF-β/transcriptional coactivator with PDZ-Binding motif (TAZ)/transcription enhancer activator domain protein (TEAD) pathways promotes collagen I production (47). Together, TGF-β-related pathway activation via various mechanisms, including advanced glycation end products (AGE)/receptor for AGE (RAGE)/high mobility group box 1 (HMGB1), results in activation of epithelial-mesenchymal transition (EMT) pathways, resulting in the elaboration of profibrotic cell types (48–50).

### Endothelium and Vascular Pathology

Diabetes-associated vascular disease affects intrarenal arteries, with afferent arteriolar matrix accumulation occurring early in DN (51). Endothelial glycocalyx degradation is linked to albuminuria (52), and its preservation with mineralocorticoid antagonism (53) and adiponectin (53) has been reported. Extraglomerular capillary proliferation (polar vasculosis) forms anastomoses between glomerular capillaries and afferent arterioles (54, 55), and extra efferent vessel development has also been documented (55, 56). Pericytic alterations, fibrointimal arterial thickening, and arteriolar hyalinosis predicts cardiovascular outcomes (57, 58). Further direct evidence of endothelial injury includes loss of endothelial fenestrations with associated podocyte injury/detachment (59). Pericytes are perivascular supporting cells with some mesenchymal stem cell properties and recent studies have demonstrated that pericyte activation, acquiring a myofibroblastic/profibrotic phenotype, can occur in the setting of peritubular capillary injury in diabetic kidneys and pericytes express sodium glucose co-transporter 2 (SGLT2), suggesting they may be a therapeutic target in DN (60–62). Juxtaglomerular hypertrophy compensates for preglomerular arteriolosclerosis/hyalinosis (63). Glomerular volume increase precedes mesangiopathic, microaneurysmal, nodular, and sclerosing changes (64). AGEs accumulate first in arterial walls, later appearing in glomerular lesions (65).

### Interstitial and Inflammatory Cell Involvement

Interstitial fibrosis initially arises from tubular collagen deposition and progresses via myofibroblast activation (66). Macrophage infiltration is a well-known feature of DN, with a potential diabetes-specific phenotype. For example, macrophages express galectin-3 and sialoadhesion, the latter in response to interleukin-1 beta (IL-1β), tumor necrosis factor-α (TNF-α), and glucose, are noted to correlate with DN stages of glomerulopathy (67, 68) and is associated with reduced thrombomodulin in glomerular endothelial cells (69). Mast cell degranulation releases cytokines, endothelins, growth factors, and proteolytic enzymes, exacerbating injury. Interstitial eosinophils, particularly aggregates of >10 per high-power field, occur in one-third of DN cases and correlate with disease severity and renal prognosis (70). Further studies on inflammatory infiltrates’ role in progressive renal injury are warranted.

## NUCLEAR HORMONE RECEPTORS, TRANSCRIPTION FACTORS AND G PROTEIN COUPLED RECEPTORS

**Figure 1.**
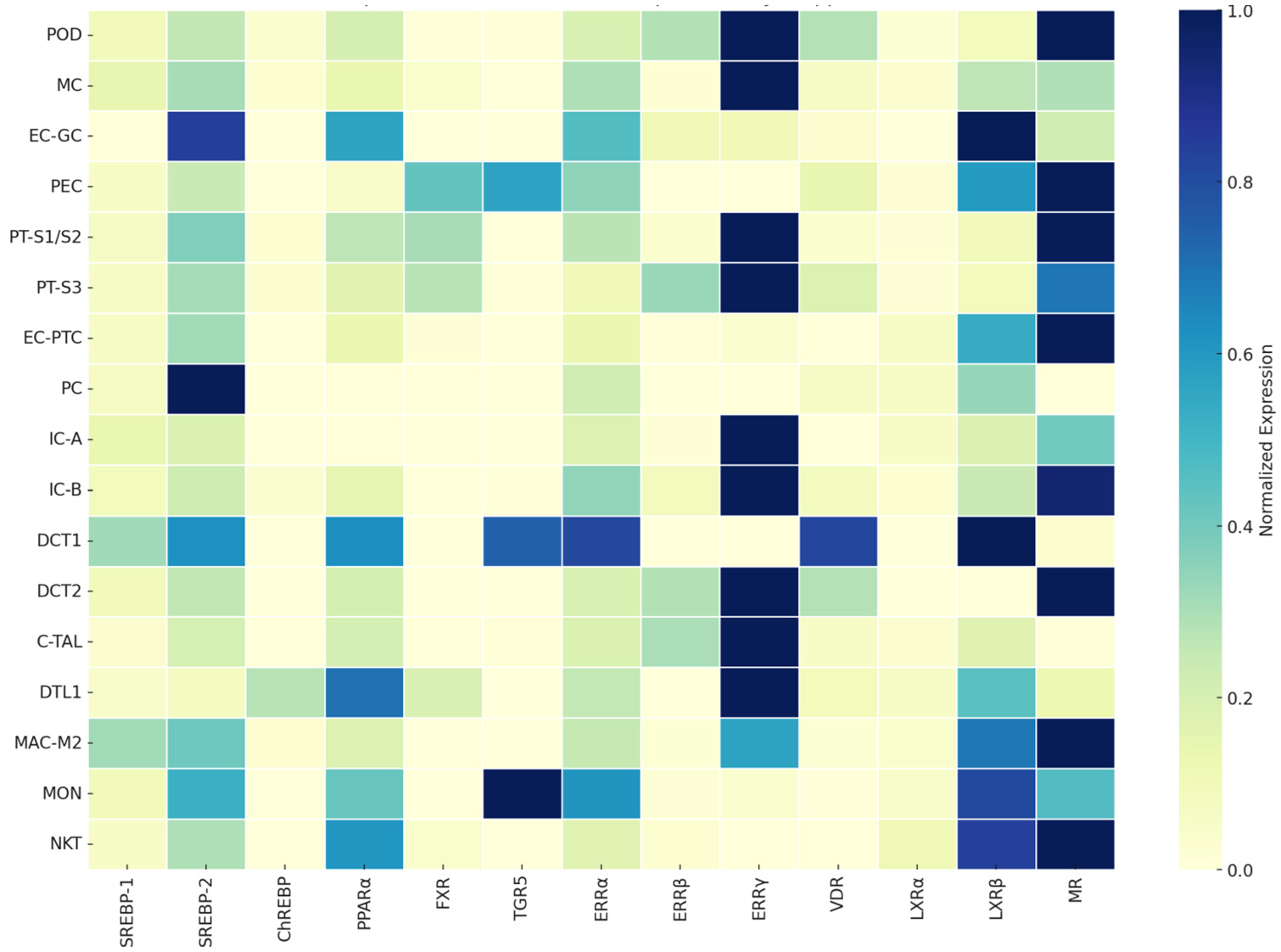
Expression of nuclear receptor, transcription factors, and GPCR transcripts in different kidney cell types based on scRNAseq of human kidneys (KPMP dataset). C-TAL, cortical thick ascending limb cell; DCT, distal convoluted tubule cell; DTL, descending thin limb cell; EC-GC, glomerular capillary endothelial cell; EC-PTC, peritubular capillary endothelial cell; IC, intercalated cell; MAC-M2, M2-macrophage; MC, mesangial cell; MON, monocyte; NKT, natural killer T cell; PC, principal cell; PEC, parietal epithelial cell; POD, podocyte; PT, proximal tubule epithelial cell.

Work in recent years has identified several nuclear hormone receptors, transcription factors, and G protein coupled receptors that act in a cell-type-specific manner across glomerular, tubular, and vascular compartments to regulate lipid metabolism, mitochondrial function, oxidative and ER stress, inflammation, cell injury, and fibrosis and thus play an important role in the pathogenesis of DKD (Fig. 1).

### The Sterol Regulatory Element-Binding Proteins

Sterol regulatory element-binding protein (SREBPs) are initially synthesized as proteins located in the endoplasmic reticulum (ER) membrane. When cholesterol levels in the cell drop or cholesterol demand increases, these proteins, along with the ER membrane protein SREBP cleavage-activating protein (SCAP), are transported to the Golgi apparatus. There, they undergo a two-step proteolytic cleavage process by site 1 and site 2 proteases, leading to the formation of the active, soluble N-terminal mature form of SREBP. This mature SREBP then translocates to the nucleus, where it binds to sterol-regulatory elements in target genes, promoting their transcription (71–73).

Two distinct genes encode SREBPs: *SREBF1*, which produces the isoforms SREBP-1α and SREBP-1c via different promoters, and *SREBF2*, responsible for encoding SREBP-2. Although SREBP-1c predominantly regulates genes involved in lipogenesis, SREBP-2 mainly controls genes related to cholesterol metabolism. SREBP-1c is widely expressed across different tissues, whereas SREBP-1α is found only in select tissues and is capable of activating genes involved in both fatty acid and cholesterol synthesis (71).

Research using transgenic mice overexpressing SREBP-1a demonstrated that increased levels of SREBP-1a in the kidney led to enhanced expression of lipogenic genes, including fatty acid synthase and acetyl-CoA carboxylase. This resulted in elevated kidney lipid accumulation, upregulation of profibrotic factors, increased urinary albumin excretion, and glomerulosclerosis (74). Further evidence for the role of SREBPs in renal lipid accumulation was provided by studies on SREBP-1c knockout mice, where high-fat diet-induced kidney lipid buildup was prevented, and proteinuria and glomerulosclerosis were mitigated compared with wild-type mice (75, 76). In addition, overexpression of glomerular SREBP-1c has been linked to alterations characteristic of diabetic kidney disease (76).

Recently, two lipogenic enzymes regulated by SREBPs, acyl-CoA synthetase short-chain family 2 (ACSS2) and fatty acid synthase (FASN), have been identified as key players in lipid accumulation, mitochondrial dysfunction, inflammation, and cellular damage in various mouse models of kidney disease, including adenine, unilateral ureteral obstruction (UUO), and diabetic db/db mice (77, 78).

SREBP-1 has been found to directly regulate transforming growth factor-beta (TGFβ) by binding to its promoter region, thereby influencing its expression and subsequent profibrotic signaling in mesangial cells. In addition, TGFβ can further stimulate SREBP-1 transcriptional activity, establishing a positive feedback loop that reinforces SREBP-1 involvement in TGFβ signaling (79).

Through its role in lipid synthesis and lipotoxicity, as well as its regulation of TGFβ-driven profibrotic signaling, SREBPs serve as a central factor in the development of obesity- and diabetes-associated kidney disease. Studies have shown that treatment with nuclear receptor FXR or vitamin D receptor (VDR) agonists in mice with diet-induced obesity and diabetes prevented kidney lipid accumulation and renal disease, at least in part by suppressing SREBP expression (80, 81).

### Carbohydrate Response Element-Binding Protein

Carbohydrate response element-binding protein (ChREBP) is a transcription factor that responds to glucose levels to regulate gene expression, influencing both glycolysis and lipogenesis (82). Recent studies have demonstrated that inducible, podocyte-specific knockdown of ChREBP in diabetic db/db mice improved proteinuria, reduced mesangial expansion, and prevented both podocyte loss and mitochondrial fragmentation (83). The beneficial effect on mitochondrial fragmentation was linked to the regulation of glyceronephosphate O-acyltransferase (Gnpat). Additional studies using whole body ChREBP knockout mice similarly reported protective effects of ChREBP inhibition in diabetic kidney disease (84, 85).

Among the transcription factors examined in the context of DKD, ChREBP has been identified as a glucose-responsive regulator of both glucose and lipid metabolism. It has been implicated in metabolic reprogramming that leads to mitochondrial dysfunction and oxidative stress (85–87). ChREBP’s role in DKD can be categorized into three main functions:

1. Regulation of lipogenesis: ChREBP activates genes involved in fatty acid synthesis and lipid storage, contributing to lipid accumulation in kidney cells (87, 88).
2. Mitochondrial dysfunction: it influences mitochondrial lipid composition and morphology, exacerbating mitochondrial dysfunction and oxidative stress in DKD (83).
3. Inflammation and fibrosis: ChREBP deficiency has been shown to reduce renal inflammation and fibrosis by inhibiting nucleotide-binding oligomerization domain-like receptor family pyrin domain-containing 3 (NLRP3) inflammasome activation (84).

Targeting ChREBP presents a promising therapeutic approach for DKD. Modulating its activity could help reduce lipid accumulation, enhance mitochondrial function, and mitigate inflammation and fibrosis in the kidneys. Future research should focus on developing molecular therapies aimed at restoring mitochondrial function and dynamics, potentially offering sustained renoprotective effects in patients with diabetes.

### Peroxisome Proliferator-Activated Receptor Alpha

Peroxisome proliferator-activated receptor alpha (PPARα) activation has demonstrated kidney-protective effects in various rodent models of chronic kidney disease (CKD), including models of type 1 diabetes mellitus (T1DM) (89), type 2 diabetes mellitus (T2DM) (90–93), diet-induced obesity (94), aging (95), obstructive nephropathy (96), and Alport syndrome (97). However, a few studies have reported only marginal or no benefit from PPARα activation (98, 99).

PPARα agonists, such as fibrates, have been evaluated in patients with type 2 diabetes in the Fenofibrate Intervention and Event Lowering in Diabetes (FIELD) study (100). However, fenofibrate’s interference with renal tubular creatinine secretion complicates the interpretation of its effects on creatinine-estimated glomerular filtration rate (99, 101). The Action to Control Cardiovascular Risk in Diabetes (ACCORD) study assessed fenofibrate’s impact on kidney function decline and renal outcomes (102, 103). Initially, patients receiving fenofibrate experienced a drop in creatinine-estimated glomerular filtration rate, likely due to changes in creatinine secretion. However, over time, the fenofibrate-treated group exhibited a significantly slower decline in estimated glomerular filtration rate compared with the control group (104). A recent clinical trial using pemafibrate, a selective PPARα activator, demonstrated an improvement in estimated glomerular filtration rate (105).

### Farnesoid X Receptor

Farnesoid X receptor (FXR) is a nuclear hormone receptor primarily found in the kidney, liver, intestines, adrenals, and vasculature. Its endogenous ligands are bile acids, with their effectiveness increasing based on the hydrophobicity of the bile acid species (106–109). Primary bile acids, such as glycine and taurine, conjugates of cholic and chenodeoxycholic acid, are produced in the liver and released into the intestines. A portion of these is converted by intestinal microbiota into secondary bile acids, including deoxycholic acid and lithocholic acid. Among these, chenodeoxycholic acid is the most potent FXR ligand.

In the kidney, FXR is predominantly located in the proximal tubules, with lesser expression in the loop of Henle and collecting ducts. Recent single-cell RNA-sequencing data from a healthy human kidney highlight the strong expression of FXR (*NR1H4*) transcripts in the proximal tubules (110, 111). Some studies have also detected FXR expression in the glomeruli. Multiple studies indicate that FXR activation is nephroprotective in various kidney disease models, including obesity, diabetes, aging, and acute kidney injury (AKI) (112–117). Activation of FXR has been shown to decrease lipogenesis, oxidative stress, inflammation, and fibrosis. The antilipogenic effects of FXR result from both reduced fatty acid synthesis and enhanced fatty acid breakdown. FXR agonism suppresses de novo renal lipogenesis by downregulating SREBP-1c expression. Mechanistically, FXR upregulates the small heterodimer partner (SHP), an orphan nuclear receptor that represses SREBP-1c expression. This FXR-induced reduction in SREBP-1c does not occur in the livers of SHP knockout mice (118). In mouse models of diet-induced obesity, FXR agonists prevent the upregulation of renal SREBP-1c, leading to reduced lipid accumulation in the kidneys (80, 115, 116). FXR activation also promotes the expression of genes involved in fatty acid catabolism in diet-induced obesity models and helps normalize mitochondrial function (80, 115, 116). Collectively, these findings suggest that FXR activation not only reduces fatty acid synthesis but also enhances fatty acid breakdown.

In diet-induced obesity models, FXR activation has been shown to increase the expression of antioxidative genes and restore glutathione metabolism, thereby reducing oxidative stress (80, 113). These protective antioxidative effects are absent in *FXR*-null mice treated with the FXR agonist INT-747, obeticholic acid. The antioxidative role of FXR is at least partly mediated by nuclear factor erythroid 2-related factor 2 (NRF2), a master regulator of the antioxidative response (80, 113). In a mouse model of ischemia-reperfusion injury, FXR activation enhanced NRF2 transcription, translation, and nuclear localization, which was correlated with increased antioxidant gene expression and reduced reactive oxygen species. In vitro knockdown experiments in hypoxic cells confirmed that this effect was NRF2-dependent (113).

Renal fibrosis, a common outcome of many kidney diseases, is reduced by FXR activation. In mouse models of diabetic nephropathy (induced by streptozotocin and a high-fat, high-cholesterol Western diet) and UUO, *FXR*-null mice exhibited more severe renal fibrosis compared with control mice (119–121). In UUO models, FXR activation decreased Smad3 expression and TGF-β1-induced YAP activity, both of which are key drivers of renal fibrosis. This effect was linked to the inhibition of YAP phosphorylation by proto-oncogene tyrosine-protein kinase Src. In addition, the antifibrotic impact of FXR activation may be partly due to the upregulation of the FXR target gene SHP (*Nr0b2*). SHP expression is reduced in UUO rats, and *SHP*-null UUO mice exhibit more severe fibrosis compared with control mice. Furthermore, renal fibrosis was alleviated in UUO rats following adenoviral SHP expression (122).

Several studies have demonstrated that FXR activation mitigates renal inflammation in kidney disease models. Various mechanisms by which FXR reduces inflammation have been identified, particularly in the liver. In hepatic cells, FXR activation directly inhibits NF-κB binding to its target promoters (119, 123). In models of diet-induced obesity, hepatic FXR becomes hyperacetylated, which disrupts FXR-mediated NF-κB inhibition (119).

Emerging research suggests that targeting intestinal FXR may have potential therapeutic benefits in obesity (124). A study found that blocking intestinal FXR with glycine-β-muricholic acid reduced ceramide levels in the intestines, thereby restoring beige fat thermogenic function. This resulted in the normalization of metabolic parameters in a diet-induced obesity model. However, these findings are somewhat contradictory to another study in which intestinal-specific FXR activation with fexaramine was found to be beneficial in obesity (125). One possible explanation for this discrepancy is that Takeda G-protein-coupled receptor 5 (TGR5) signaling may have been activated in the latter study, albeit not directly by fexaramine, leading to the observed metabolic benefits.

### Takeda G-Protein-Coupled Receptor 5

The G-protein-coupled bile acid receptor 1, also known as Takeda G-protein-coupled receptor 5 (TGR5), is a G-protein-coupled receptor that is activated by bile acids. Upon activation, TGR5 signals through the G-α pathway, leading to an increase in cyclic adenosine monophosphate levels. Unlike FXR, TGR5 is most effectively activated by lithocholic acid and taurine-conjugated lithocholic acid (126). Ursodeoxycholic acid, which is FDA-approved for treating primary biliary cholangitis, has also been found to weakly activate TGR5 (127). TGR5 is expressed in several organs, including the kidney, liver, gallbladder, intestine, adipose tissue, and central nervous system. In the kidney, TGR5 is present in both the tubules and glomeruli of mice and humans, with a higher expression in the tubules (128).

Renal TGR5 expression is reduced in kidney biopsy specimens from diabetic and obese patients. In addition, in patients with diabetes, renal TGR5 expression is directly correlated with estimated glomerular filtration rate and inversely correlated with proteinuria, global sclerosis, and the rate of estimated glomerular filtration rate decline (128). Studies have shown that TGR5 activation provides renal protection in models of diet-induced obesity, diabetes, aging, and ischemia-reperfusion injury (113, 128–130). The protective effects of TGR5 activation include enhancing mitochondrial biogenesis while reducing oxidative stress and inflammation. In a diet-induced obesity model, the TGR5-specific agonist INT-777 [6α-ethyl-23(S)-methylcholic acid] promoted kidney protection by increasing mitochondrial bio-genesis via upregulation of PPARγ-coactivator-1α (PGC-1α), a key regulator of mitochondrial biogenesis. INT-777 also reduced oxidative stress by upregulating superoxide dismutase 2 and restoring sirtuin 3 expression, a mitochondrial deacetylase that activates superoxide dismutase 2 and is downregulated in diet-induced obese mice (128). These findings were further validated in TGR5 knockout mice. In these mice, PPARγ-coactivator-1α and sirtuin 3 expression levels were reduced compared with control mice, and treatment with INT-777 did not restore renal expression of these proteins (128).

Although INT-777 treatment has been shown to reduce inflammation in diet-induced obese mice (128), the most compelling mechanistic insight into TGR5’s role in inflammation regulation was demonstrated in a diabetic nephropathy model (113). In this model, TGR5 activation suppressed NF-κB activity by stabilizing NF-κB inhibitor α (IkBα). This stabilization occurred due to enhanced interaction between IkBα and β-arrestin, leading to reduced inflammation in the kidney.

### Estrogen-Related Receptors Alpha, Beta, and Gamma

The estrogen-related receptors (ERRs), including estrogen-related receptor alpha (ERRα) (*NR3B1*, *ESRRA* gene, Chr. 11q12), estrogen-related receptor beta (ERRβ) (NR3B2, ESRRB gene, Chr. 14q24.3), and estrogen-related receptor gamma (ERRγ) (*NR3B3, ESRRG* gene, Chr. 1q41), are members of the nuclear receptor family. These receptors are considered orphan receptors, as no definite endogenous ligands have been identified. However, recent studies have identified several agonists capable of activating ERRs (131–137). Both ERRα and ERRγ are highly expressed in the kidneys of mice and humans. Notably, ERRs do not bind natural estrogens and do not participate in classical estrogen signaling pathways (138–140). ERRα and ERRγ are strongly activated by PGC-1α and PGC-1β, whereas RIP140 and NCoR1 serve as key corepressors of ERR activity. In addition, ERRs undergo posttranslational modifications, such as phosphorylation, sumoylation, and acetylation, which influence their DNA binding and recruitment of coactivators (141–143). ERRα and ERRγ regulate the transcription of genes involved in mitochondrial biogenesis, oxidative phosphorylation, the tricarboxylic acid (TCA) cycle, fatty acid oxidation, and glucose metabolism (140). Although these two isoforms share overlapping gene activation, their effects may differ based on interactions with corepressors, coactivators, posttranslational modifications, or variations in cellular expression (140, 144). Contrasting effects of ERRα and ERRγ have been observed in breast cancer (145, 146), liver gluconeogenesis (144, 146), skeletal muscle function (144, 146), macrophage activity (119, 141, 144), and lactate dehydrogenase A (LDHA) regulation in anaerobic glycolysis (140).

Whole body ERRα knockout mice exhibit low blood pressure, hypernatremia, hypokalemia, and hyperreninemia (141). In these mice, genes involved in renal sodium and potassium regulation (*Scnn1a, Atp1a1, Atp1b1*), Bartter syndrome-related genes (*Bsnd, Kcnq1*), systemic blood pressure control genes (*Ghr, Gcgr, Lepr, Npy1r*), and renin-angiotensin-aldosterone system (RAAS) genes (*Ren1, Agt, Ace2*) are differentially expressed (141). Conversely, whole body ERRγ knockout mice develop severe hyperkalemia (147). A recent study using ERRγ floxed mice crossed with Sim1-Cre mice, which mediates recombination in renal and nonrenal tissues, found that ERRγ plays a crucial role in regulating mitochondrial oxidative phosphorylation (OxPhos) and fatty acid oxidation (FAO) in renal tubules. In addition, ERRγ cooperates with hepatocyte nuclear factor 1β (HNF1β) to regulate renal tubular transport, including SGLT2. In patients with chronic kidney disease (CKD), ERRγ expression in renal tubules was found to correlate with glomerular filtration rate (GFR) and share several common genes (148). ERRα activation has been shown to provide protection against cisplatin-induced acute kidney injury (AKI) (149), whereas ERRγ activation has been found to protect against puromycin aminonucleoside (PAN)-induced podocyte apoptosis (150). Conversely, whole body ERRα knockout mice displayed worsened folic acid-induced kidney disease (151).

### Vitamin D Receptor

The VDR is a nuclear hormone receptor predominantly expressed in the kidney, intestine, parathyroid gland, bronchi, bone, and thymus, though it is also found in other tissues (152). Within the kidney, VDR expression is highest in the distal tubules (110, 111), with lower levels observed in the proximal tubules, macula densa, glomerular parietal epithelial cells, and podocytes (153).

Studies have demonstrated that kidney disease is more severe in *VDR*-null mice than in wild-type mice across various models, including T1DM (154, 155), UUO (156), and cisplatin- and lipopolysaccharide-induced AKI (157, 158). In addition, renal VDR expression was found to be reduced in UUO models (159). Importantly, VDR activation has been shown to be nephroprotective in multiple conditions, including diet-induced obesity (81), T1DM (155), type 2 diabetes mellitus (T2DM) (160, 161), UUO (159), Alport syndrome (162), 5/6 nephrectomy (163), and various AKI models (157, 164, 165). Collectively, these studies indicate that VDR protects the kidney by reducing RAAS signaling, preventing lipotoxicity, and maintaining the integrity of the glomerular basement membrane.

VDR activation has been found to be beneficial in diet-induced obesity models. Compared with mice fed a low-fat diet, those on a high-fat diet exhibited increased renal lipid accumulation, fibrosis, inflammation, and RAAS activation, all of which were mitigated by VDR agonist treatment. Notably, VDR activation reduced SREBP-1c and SREBP-2 expressions, along with their target genes responsible for de novo fatty acid and cholesterol synthesis, potentially through upregulation of FXR. In addition, VDR activation increased PPARα and decreased CD36, suggesting that it enhances fatty acid oxidation while reducing fatty acid uptake. These findings highlight VDR-mediated lipid reduction as a novel mechanism underlying its renoprotective effects in diet-induced obesity (81). Furthermore, these results suggest significant interactions between several nuclear receptors discussed in this review.

In a T1DM model, nephrin expression was reduced in *VDR*-null mice compared with wild-type controls, whereas calcitriol treatment was found to upregulate nephrin expression in cultured podocytes (155). Increased nephrin expression following calcitriol treatment was also observed in vivo in T1DM (166) and T2DM models (161). Vitamin D activation further protected mice with podocyte-specific VDR overexpression from T1DM-induced kidney damage. Similarly, overexpression of VDR in podocyte-specific VDR-null mice conferred protection from T1DM. Collectively, these findings strongly suggest that VDR plays a critical role in preserving glomerular podocytes.

### Liver X Receptor Alpha and Beta

Liver X receptor alpha (LXRα) (*Nr1h3*) and liver X receptor beta (LXRβ) (*Nr1h2*) are key regulators that enhance cholesterol efflux and suppress inflammation. Studies in mice have shown that LXRα/β double knockout mice exhibit increased urinary albumin excretion, kidney cholesterol accumulation, and elevated inflammation, even without induced diabetes. These pathological changes are significantly exacerbated when diabetes is induced via streptozotocin administration (167). Conversely, treating diabetic mice with the selective LXR agonist *N*, *N*-dimethyl-hydroxycholenamide (DMHCA) led to reductions in urinary albumin and nephrin excretion, glomerular mesangial expansion, and levels of cholesterol and triglycerides in both plasma and the glomeruli. In addition, DMHCA treatment reduced inflammatory, oxidative stress, and profibrotic markers in the kidney (167). These findings have been corroborated by other studies (168). Notably, transgenic overexpression of LXRα in macrophages significantly mitigated renal disease in LDL receptor knockout mice with streptozotocin-induced diabetes (169).

Although studies on LXR agonists in human patients with diabetes remain limited, a major concern is that LXR activation also induces SREBP-1 and ChREBP, which promote lipogenesis (170). However, unlike conventional LXR agonists, DMHCA does not trigger lipogenesis due to its structural similarity to desmosterol (171, 172). Similar to desmosterol, DMHCA enhances cholesterol efflux while simultaneously reducing inflammation (173, 174). Future research into DMHCA or other desmosterol-modulating agents may pave the way for novel therapeutic strategies to treat diabetic kidney disease.

### Mineralocorticoid Receptor

The mineralocorticoid receptor (MR) is a family member of steroid nuclear receptors activated by mineralocorticoids like aldosterone and regulates gene expression involved in electrolyte and fluid balance. In the kidney, MR is expressed in distal tubules, collecting duct, podocytes, fibroblasts, and mesangial cells (175). Although primarily known for its role in the kidneys, MR is also present in other tissues, including the heart, vasculature, and immune cells. In diabetic kidney, MR activation contributes to inflammation, oxidative stress, and fibrosis, accelerating kidney damage. Excessive aldosterone and MR overactivation in diabetes worsen glomerular injury, proteinuria, and renal fibrosis, leading to disease progression (176, 177). MR antagonists (MRAs), such as spironolactone, eplerenone, and finerenone, have been studied in mouse models of DKD and show protective renal effects.

Mineralocorticoid receptor (MR) antagonists have demonstrated significant renoprotective effects in various mouse models of DKD. In STZ-induced diabetic rats, spironolactone administration for 3 wk reduced renal collagen deposition in the glomerular, tubulointerstitial, and perivascular areas (178). Also, MR inhibition lowered oxidative stress markers, preventing tubular apoptosis. Another study demonstrated in diabetic db/db mice and stz-induced diabetic rats that administration of eplerenone treatment reduced albuminuria, preserve the podocyte structure, glomerular mesangial expansion, and hypertrophy associated with reduced inflammation (179). In high salt-induced uninephrectomy (UNx) diabetic db/db mice with, nonsteroidal antagonist finerenone administration ameliorated the kidney injury by normalizing the blood pressure and prevented glomerular injury, nodular and insudative lesions. Furthermore, finerenone decreased expression of fibronectin and periglomerular macrophage accumulation (180). Recent study indicated that nonsteroidal antagonist finerenone treatment preserved mitochondrial dysfunction and reduced the mitoROS production and tubular cell apoptosis in high-fat diet/stz-induced diabetic mice (181). All this evidence suggests, MR antagonism provides renoprotective effects by reducing proteinuria, inflammation, fibrosis, oxidative stress, and podocyte injury. These findings support the use of MRAs as potential therapeutic agents to slow kidney disease progression in diabetes.

## ROLE OF LIPID METABOLISM IN DKD

**Figure 2.**
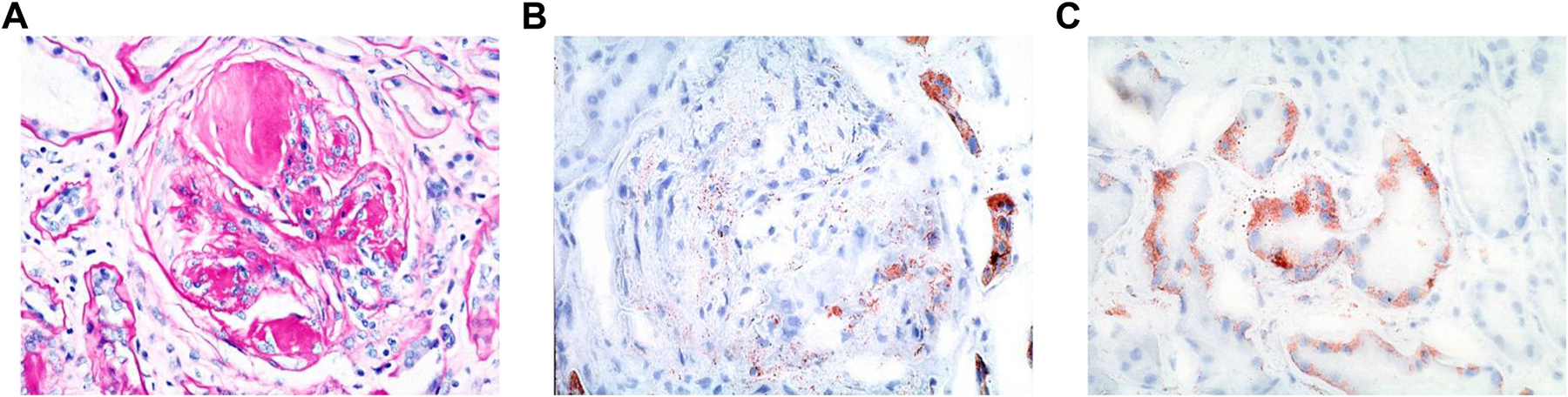
In human diabetic kidneys, Kimmelstiel–Wilson nodules, a hallmark of diabetic nephropathy, as shown by Periodic Acid–Schiff (PAS) staining (*A*), are associated with neutral lipid droplets highlighted by Oil Red O staining in glomeruli (*B*) and surrounding tubulointerstitial regions (*C*).

**Figure 3.**
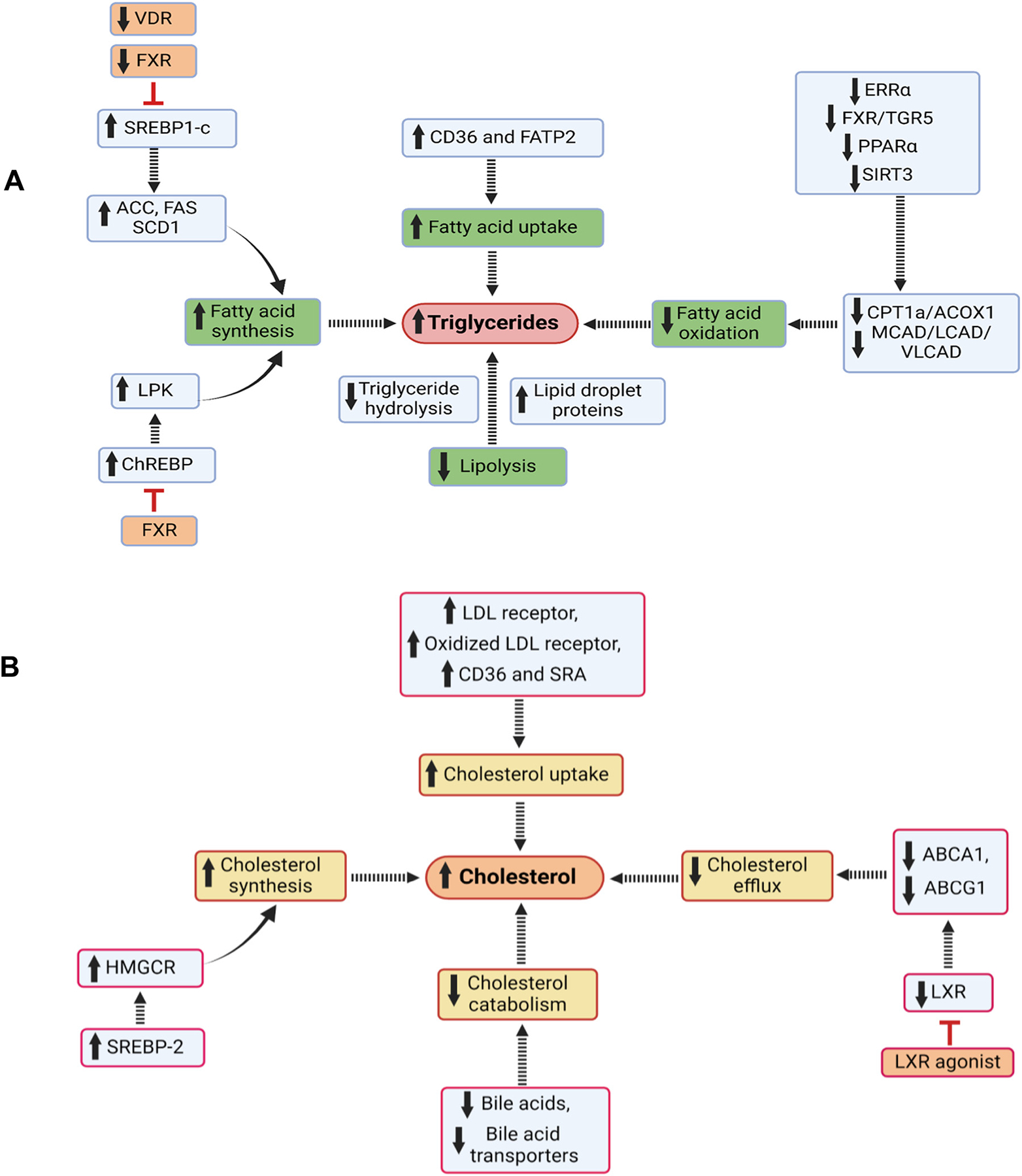
Mechanisms and pathways that regulate triglyceride (*A*) and cholesterol metabolism (*B*) and accumulation in the kidney.

It has long been established that hyperlipidemia and increased renal accumulation of triglycerides and cholesterol are associated with diabetic pathology (Fig. 2–3), but the specific role of different lipid species is the subject of continuous research.

### Cholesterol

Early studies focused on the relationship between free fatty acids (FFAs) and insulin resistance (182–184). After publication of the Randle cycle in 1963, described the biochemical process by which glucose and fatty acid oxidation compete for oxidation and metabolic uptake (185). More recently, an association between cholesterol and insulin resistance was discovered and has been studied extensively in the 21st century, which coincides with an established correlation between elevated cholesterol and negative outcomes in diabetic nephropathy in human patients (186, 187).

In 2007, a study revealed the direct effect of cholesterol on insulin signaling and β-cell dysfunction (188). This study used apoE-deficient mice to circumvent the issue of elevated FFAs coinciding with traditional elevation of cholesterol using a high-fat diet. Cholesterol was further elevated by breeding apoE-deficient mice onto an ob/ob genetic background. The mouse models used in this study also show an increased triglyceride level relative to controls, so isolated pancreatic islets and cultured β-cells were used to study direct effects of cholesterol. This study successfully showed an inverse relation between serum cholesterol and insulin secretion, and that cholesterol depletion restored normal insulin secretion. Further, they showed how excess cholesterol increases neuronal nitric oxide synthase dimerization to inhibit insulin secretion.

Additional studies have shown that cholesterol efflux is impaired in patients with diabetic nephropathy compared with age-matched controls by comparing the ability of each patient’s serum to induce cholesterol efflux by ATP-binding cassette transporter A1 (ABCA1) and scavenger receptor class B type I (SR-BI) in cell culture (189). All patients with diabetes saw a decrease in SR-BI-mediated efflux, but only those with microalbuminuria or proteinuria showed impairment in ABCA1-mediated efflux.

This result was elaborated upon by another study that showed that ABCA1 is downregulated in normal human podocytes upon exposure to sera from patients with type 1 diabetes and albuminuria (DKD) and in biopsies of glomeruli from patients with DKD compared with donor controls. This study also showed that cholesterol efflux induced by cyclodextrin treatment prevented podocyte injury after serum exposure, but cholesterol synthesis inhibition by simvastatin did not (190). Several studies have shown that cyclodextrin improves outcomes in mouse and cell culture models of diabetic kidney disease (190, 191).

LXR agonists have also been used to increase ABCA1 expression since it was established that decreased LXR expression coincides with decrease in ABCA1 in diabetic nephropathy (192). LXR agonists have been shown to promote ABCA1-mediated cholesterol efflux and improve outcomes in mouse and cell culture diabetic kidney disease models (167, 169, 193–197). It has also been published that Tangshen formula, a traditional Chinese medicine that has been used in patients with diabetic kidney disease to reduce plasma lipids and proteinuria and improve estimated glomerular filtration rate (eGFR), may work by promoting ABCA1-mediated renal cholesterol efflux (198).

A new class of small-molecule drugs to increase ABCA1-mediated cholesterol efflux was described in a 2021 article (168, 199). These are 5-arylnicotinamide compounds that upregulate ABCA1 and its associated cholesterol efflux by targeting oxysterol binding protein like 7 (OSBPL7) and were shown to normalize proteinuria and prevent decline in renal function in adriamycin-induced nephropathy and Alport syndrome mouse models of kidney disease. Interestingly, OSBPL7 deficiency was later implicated in podocyte injury in glomerular disease in a robust study using mouse models, cell culture, and zebrafish (200). This study showed that ER stress drives apoptosis in OSBPL7-deficient podocytes. Previous work by this same group identified a mechanism by which local TNF drives free cholesterol-mediated podocyte injury, independent of circulating TNF, tumor necrosis factor receptor (TNFR)1, or TNFR2 levels. TNF induces apoptosis in podocytes via NFATc1-mediated repression of ABCA1 and inhibition of SOAT1, leading to cholesterol imbalance. In vivo experiments confirmed that TNF-induced albuminuria is exacerbated in podocyte-specific ABCA1-deficient mice but is mitigated by cholesterol depletion (201).

### Sphingolipids

**Figure 4.**
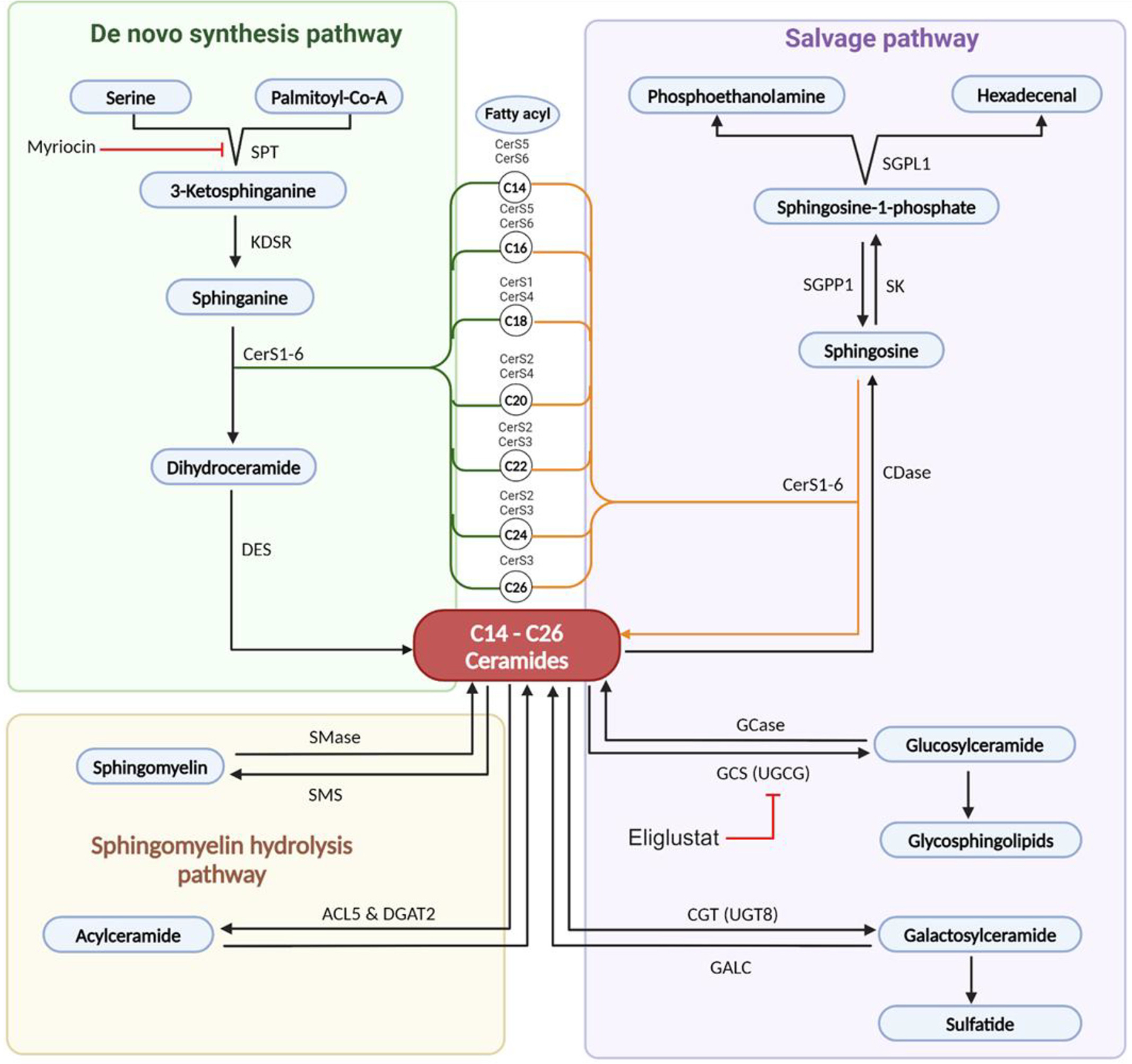
Mechanisms and pathways that regulate sphingolipids metabolism.

Once thought to be passive cell membrane barrier molecules, sphingolipids have recently been identified as a class of bioactive lipids that play a significant role in biochemical signaling in diabetic nephropathy (202). Ceramides, a type of sphingolipid, can be synthesized via three primary pathways, including de novo synthesis in the endoplasmic reticulum (ER), the sphingomyelinase pathway in which sphingomyelin is hydrolyzed to ceramide via acid or neutral sphingomyelinases, and a salvage pathway, which breaks down more complex sphingolipids to sphingosine, which can be reacylated in the ER. Ceramides can be further metabolized in the Golgi apparatus to produce additional sphingolipids, including sphingosine (when ceramides are deacylated by a specific ceramidases), glucosylceramide (when glucosylceramide synthase adds a glucose moiety to ceramides), and sphingomyelins (sphingomyelin synthases adds a phosphocholine headgroup to ceramides). Ceramides and sphingosine can both be phosphorylated by specific kinases to produce sphingosine-1-phosphate and ceramide-1-phosphate, which each have unique signaling properties (203) (Fig. 4).

Ceramides have established roles in triggering inflammation, ER and oxidative stress, and apoptosis (204–207). Previous research suggests involvement of ceramides in the pathogenesis of diabetic kidney disease, including documentation of elevated levels of ceramide in human patients with obesity, insulin resistance, and diabetic nephropathy (205, 208–212). Observational studies show that patients with elevated serum ceramide levels tend to have more severe insulin resistance (209, 213), and it has also been shown that insulin-resistant patients also tend to have higher levels of ceramide in skeletal muscle (208, 214). Sphinganine, a ceramide precursor, and sphingosine, which is produced by the breakdown of ceramides, have also been detected at higher levels in plasma from patients with type 2 diabetes relative to controls (215).

Ceramides can contribute to the development of insulin resistance, and studies have shown that inhibition of de novo ceramide synthesis can prevent insulin resistance in mouse models and cell culture (216, 217). Ceramides may contribute to diabetic pathologies via TLR4-mediated insulin resistance (218, 219) and increasing pancreatic β-cell dysfunction and apoptosis (220, 221). Ceramides are less well-studied in the specific context of diabetic kidney disease, but some studies have shown that certain long-chain ceramides and glucosylceramides are increased in patients and mouse models of diabetic kidney disease (db/db), whereas very long-chain ceramides are decreased in diabetic kidney tissue (222–224). A 2022 study showed decreased ceramide synthesis and milder tubulointerstitial injury in *Cfb*-knockout diabetic mice, and cell culture studies in HK-2 cells under high glucose conditions validated that ceramide synthesis inhibition rescued cell injury (225). Levels of ceramide synthase 6 (CERS6) expression are upregulated in renal cortexes of db/db mice, and decreased expression was associated with restoration of PINK1-mediated mitophagy, improved mitochondria, and mitigation of kidney fibrosis typically seen in diabetic kidney disease that may be due in part to abnormal ceramide accumulation (226). The link between ceramides and fibrosis, mitochondrial dysfunction, and podocyte damage has been documented in the context of chronic kidney disease (227).

Glycosphingolipids, consisting of carbohydrate groups glycosidically bound to ceramide bases, have been studied in the context of kidney pathology in type 1 Gaucher disease, a lysosomal lipid storage disorder. It has been shown that excess glycosphingolipids, including glucosylceramide and ganglioside GM3, are associated with insulin resistance in Gaucher disease (228, 229). In mouse models, glycosphingolipids hexosylceramides (HexCers) and lactosylceramides (LacCers) have been shown to be elevated in kidney cortex sections, and the same study showed that HexCers levels are also higher in mesangial and glomerular cells grown in high glucose in vitro (230). This study concluded that inhibition of glucosylceramide synthase reversed hypertrophy in mesangial cells through decreases in pAkt and pSmad3 and increased protein degradation, implying that the accumulation of glycosphingolipids HexCers and LacCers in mesangial and glomerular cells plays a role in the development of diabetic kidney pathologies (230).

Sphingosine 1-phosphate (S1P) and ceramide 1-phosphate (C1P) are two well-studied sphingolipid metabolites. S1P in particular has been extensively studied and reviewed in the context of diabetic kidney disease (231–233). S1P binds to five G-protein-coupled receptors (S1P1–S1P5) to produce its effects. S1P1 activation has been shown to be protective in models of acute kidney injury (234–236) and more recently in rat, mouse, and cell culture models of diabetic nephropathy (237–239). By contrast, inhibition rather than activation of the S1P2 receptor has been shown to mitigate inflammation and fibrosis in diabetic rat kidneys (240). S1P receptors S1P3-S1P5 remain underexplored in the context of diabetic kidney disease, but evidence suggests that they may also be involved in regulating renal inflammation and fibrosis (241). Several drugs targeting S1P receptors have already been FDA approved for the treatment of multiple sclerosis and autoimmune conditions. With further evidence, these drugs could have translational value in treating DKD as well.

Ceramide-1-phosphate (C1P)’s role in DKD is an area of active research, with several studies suggesting its involvement in key pathological mechanisms. C1P may play a protective role in DKD; it has been found that elevated levels of sphingomyelin phosphodiesterase acid-like 3 b (SMPDL3b) in diabetic podocytes causes dysfunction in insulin signaling and decreases in C1P. Restoration of C1P levels stopped the progression of DKD in a db/db mouse model (242). It has also been shown that SMPDL3b interacts with ceramide kinase (CERK), the enzyme responsible for converting ceramide to C1P, in differentiated human podocytes, influencing C1P production (243).

## ROLE OF IMPAIRED MITOCHONDRIAL FUNCTION IN DKD

Mitochondrial dysfunction, particularly the disruption of oxidative phosphorylation (OXPHOS) and associated oxidative stress has emerged as a unifying factor in the onset and advancement of DKD (244–246) There is increasing interest in therapeutic strategies aimed at mitigating mitochondrial impairment, which plays a central role in DKD pathogenesis by causing energy deficits, reactive oxygen species (ROS) accumulation, and cellular stress. Traditional therapies, while stabilizing kidney function, fall short of fully halting DKD progression. Among all the factors, mitochondrial dysfunction is the most “Unifying factor” for DKD and is essential for elucidating the role of mitochondrial oxidative stress in the onset and progression of DKD. As a high-energy-demanding organ, the kidney relies heavily on OXPHOS; however, in DKD, hyperglycemia induces a hypoxic state that inhibits OXPHOS, pushing cells to increase glycolysis to generate ATP rapidly. Although this shift compensates for energy deficits, it inadvertently causes damage to kidney cells (247, 248). Furthermore, abnormalities in FAO and amino acid metabolism result in the accumulation of lipids and uremic toxins, leading to renal cell stress and apoptosis (249, 250). Reduced FAO and lipid accumulation in kidney cells from patients with DKD have been identified as key metabolic disturbances (251). These lipid abnormalities contribute to mitochondrial dysfunction by disrupting mitochondrial dynamics, increasing ROS production, and impairing energy production.

### Mitochondrial Fusion and Fission

Mitochondria are highly dynamic organelles with the capacity for continuous shape and size alterations. This plasticity enables mitochondria to adapt to changing cellular energy demands, stress conditions, and metabolic states (252–254). Through the processes of fusion and fission, mitochondria can merge to form elongated networks or divide into smaller units, which supports efficient energy distribution, quality control, and removal of damaged mitochondria (254, 255). This adaptability is essential for maintaining cellular health and is particularly important in tissues with high energy requirements, such as the kidneys. In molecular studies, mitochondrial fusion is regulated by the proteins mitofusin 1 (MFN1), mitofusin 2 (MFN2), and optic atrophy protein 1 (OPA1). MFN1 and MFN2 are embedded in the outer mitochondrial membrane, whereas OPA1 is localized within the inner membrane (256). The fusion process is initiated by interactions between MFNs on adjacent mitochondria and coordinated with OPA1, which together reduce mitochondrial fragmentation and promote elongation, as demonstrated by an increase in the aspect ratio (AR) (257). Conversely, mitochondrial fission is primarily governed by dynamin-related protein 1 (DRP1), mitochondrial fission protein 1 (Fis1), and mitochondrial fission factor (MFF) (258).

DRP1, a key cytosolic mediator of fission, is activated and subsequently translocated to the mitochondrial outer membrane, where it interacts with MFF and Fis1 (259). Dysregulation of these processes can result in excessive mitochondrial fission, promoting the release of cytochrome c from mitochondria and contributing to end-organ damage. The activity of DRP1 is modulated by various posttranslational modifications, such as phosphorylation, ubiquitination, sumoylation, and S-nitrosylation (260, 261). Dysregulation of DRP1 can result in excessive mitochondrial fission, which promotes the release of cytochrome c from mitochondria, a process associated with end-organ damage. This interaction forms a constriction ring-like structure around the mitochondrion, enabling its division.

Mitochondrial fission is increased in kidneys of diabetic db/db mice (262). The study showed that the mouse model with podocyte-specific deletion of Drp1, when crossed with db/db diabetic model, showed protective effects against hallmark features of diabetic nephropathy, including reduced albuminuria, decreased mesangial matrix expansion, and improved podocyte foot process architecture, compared with diabetic mice with intact Drp1 (262, 263). It has been demonstrated that cultured podocytes from Drp1-deficient mice exhibited elongated mitochondria, with oxygen consumption rates restored to control levels. In parallel, pharmacologic inhibition of Drp1 using mitochondrial division inhibitor 1 (Mdivi-1) resulted in podocytes with healthier mitochondria and conferred protection against diabetic nephropathy progression (264). These findings underscore the importance of Drp1 and mitochondrial fission in preserving mitochondrial integrity and energy production in the kidneys. The regulation of Drp1 via phosphorylation is crucial for its mitochondrial translocation, influencing fission activity in cell-specific contexts. The posttranslational regulation of Drp1, particularly through phosphorylation, is crucial for its translocation to mitochondria (258). Several kinases modulate Drp1 subcellular localization by phosphorylating conserved serine residues, primarily influencing mitochondrial dynamics. Notably, recent work demonstrated that phosphorylation of Drp1 at Ser637 in humans (or Ser600 in mouse Drp1 isoform b) promotes mitochondrial fission in podocytes under high glucose conditions. In alignment with these results, another study found that Ca^2+^/calmodulin-dependent protein kinase Iα (CaMKIα) phosphorylation of Drp1 at this conserved site enhances Drp1’s recruitment to mitochondria. In contrast, other studies suggest that protein kinase A-mediated phosphorylation of Drp1 at Ser600 reduces its GTPase activity. These conflicting findings imply that phosphorylation effects at this site may vary depending on cell type and specific stimuli. Recently, it has been demonstrated that mitochondrial morphology like ruptured outer membrane and inner membrane cristae fragmentation, occurs in proximal tubules of db/db mice. In addition, mitochondrial complex I activity is decreased in the kidneys of diabetic mice. Pharmacological administration of nicotinamide riboside (NR) to diabetic mice prevented those changes in mitochondrial morphology and function (265). A recent study highlighted the role of NDUFS4, a gene that encodes an accessory subunit of mitochondrial complex I, [also known as NADH dehydrogenase (ubiquinone) iron-sulfur protein 4, mitochondrial] in regulating cristae remodeling in DKD, demonstrating that NDUFS4 plays a crucial role in regulating cristae morphology (the structure of the inner mitochondrial membrane) in renal podocytes (266). Furthermore, by overexpressing NDUFS4 in diabetic mice, the authors noticed significant improvements in mitochondrial dynamics and cristae morphology. The study also found that diabetic mice with podocyte-specific overexpression of NDUFS4 exhibited reduced albuminuria, indicating improved kidney function. The researchers used advanced techniques like proximity labeling and super-resolution imaging to identify the role of the cristae shaping protein STOML2 in linking NDUFS4 with improved cristae morphology (266). These findings contribute to a better understanding of the molecular mechanisms underlying DKD and highlight the importance of mitochondrial complex assembly and overall mitochondrial function in kidney health, suggesting a promising therapeutic role of NDUFS4 in slowing the progression of DKD. Overall, these insights indicate that targeting mitochondrial dynamics holds therapeutic promise for improving mitochondrial morphology and fitness in DKD. New targets and drugs addressing various components of mitochondrial dynamics may soon emerge to address DKD and other diseases involving mitochondrial dysfunction. However, extensive research is still needed to fully harness mitochondrial dynamics as a viable target for DKD therapy.

### Mitophagy

Impaired mitophagy, the selective degradation of damaged mitochondria via autophagy, as a critical factor in DKD pathogenesis. Reduced mitochondrial DNA, accumulated mitochondrial damage, ROS production, and decreased ATP levels worsen glomerular and tubular cell damage, underscoring mitophagy’s importance in maintaining kidney cell function (267). Mitophagy is orchestrated by key pathways, including the PINK1/Parkin pathway and receptors such as BCL2 interacting protein 3 (BNIP3), BNIP3-like protein (BNIP3L), and FUN14 domain containing 1 (FUNDC1), which target damaged mitochondria for autophagic clearance. Dysregulation of mitophagy in DKD disrupts this quality control, exacerbating cellular stress and disease progression, making it a promising target for therapeutic intervention in DKD. Reduced mitophagy in renal tubular epithelial cells (RTECs) and podocytes contribute to mitochondrial dysfunction, inflammation, and cell damage (268). Key regulators like PINK1 and Parkin, crucial for mitochondrial quality control, show reduced expression, leading to increased ROS and cellular injury. Studies indicate that proteins such as OPTN can counteract these effects, promoting mitophagy and reducing mitochondrial ROS, while loss of OPTN exacerbates inflammation. Other factors, including PGRN, FoxO1, and lncRNA SNHG17, are implicated in PINK1/Parkin-mediated mitophagy, and disruptions in their pathways contribute to DKD progression (269, 270). Thus, targeting mitophagy could be critical for mitigating DKD. Growing evidence highlights the role of mitophagy in DKD pathogenesis. In rat mesangial cells, autophagy markers LC3I/LC3II and p62 showed time-dependent expression changes, with increased Parkin recruitment to damaged mitochondria and enhanced autophagic vacuole formation. In renal mesangial cells, high-glucose conditions reduced autophagy-related proteins like Beclin1, PINK1, and Parkin (271). Stimulating mitophagy via the mTOR/PINK1/Parkin and BNIP3/BNIP3L pathways improves renal inflammation and glomerular integrity. In glomerular mesangial cells, the PI3K/AKT/mTOR pathway modulates autophagy, where inhibitors such as triptolide and ursolic acid can restore autophagy and reduce cell damage. In endothelial cells, mitophagy activators, like Torin1, and Nrf2/ARE signaling alleviate mitochondrial dysfunction and protect renal function in diabetic models, suggesting mitophagy’s therapeutic potential in DKD (272, 273).

Another study investigated the therapeutic effects of Astragalin, a flavonoid from *Thesium chinense Turcz*., on renal injury in streptozotocin (STZ)-induced diabetic mice (274). It shows that AG treatment improves proteinuria and reduces renal damage without affecting blood glucose levels by suppressing aldose reductase (ALR2) activity and oxidative stress. Astragalin modulates mitochondrial quality control, reducing apoptosis, enhancing mitochondrial biogenesis, maintaining mitochondrial dynamics, and improving energy metabolism. In high glucose and lipids-stimulated HK2 cells, astragalin activates the AMP-activated protein kinase (AMPK)-PGC1α pathway, essential for mitochondrial function, suggesting astragalin’s potential as a treatment for diabetic kidney disease (274).

An additional recent study highlights the role of the Meteorin-like (Metrnl) gene in addressing diabetic kidney disease (275). It shows that Metrnl expression is reduced in renal tubules under diabetic conditions and is inversely correlated with DKD pathological changes. Administering recombinant Metrnl (rMetrnl) or overexpressing Metrnl reduces lipid accumulation, alleviates renal injury, and maintains mitochondrial homeostasis in diabetic mice (275). The beneficial effects are mediated through the Sirt3-AMPK signaling axis, promoting mitochondrial function and thermogenesis. This research suggests that Metrnl could be a promising therapeutic strategy for treating DKD by regulating lipid metabolism and mitochondrial function (275).

### Role of Lipids in Impaired Mitochondrial Function in DKD

In DKD, the harmful effects of excessive lipid accumulation in tissues (lipotoxicity), play a crucial role in the progression of the disease. In diabetes, both high blood sugar and altered lipid metabolism contribute to the accumulation of toxic lipid species, including ceramides, in kidney cells. The kidneys, due to their high metabolic demands, are particularly susceptible to lipid overload, which exacerbates renal damage (276, 277). Circulating FFAs, often elevated in conditions like obesity and insulin resistance, overwhelm the kidney’s ability to metabolize lipids effectively. This results in lipid buildup in renal cells, especially podocytes and tubular epithelial cells (TECs), leading to mitochondrial dysfunction, renal steatosis, and impaired kidney function (276, 277). The accumulation of ceramides in these cells intensifies lipotoxicity, driving inflammation, fibrosis, and further cellular damage. Although ceramides are important for normal cellular functions like membrane structure and signaling, excessive accumulation in the kidney disrupts cellular homeostasis. In diabetic conditions, ceramide production is upregulated, particularly in the kidneys, contributing to harmful lipid overload. This accumulation activates proinflammatory and profibrotic pathways, induces oxidative stress, and damages mitochondrial function. The resulting dysfunction in mitochondria is key energy producers in cells, impairs kidney function by promoting oxidative stress and triggering cell death (276, 277). These processes worsen glomerulosclerosis, tubulointerstitial fibrosis, and ultimately kidney failure.

Impaired mitochondrial function is a central feature of lipotoxicity in DKD. Lipid overload disrupts mitochondrial dynamics, leading to impaired oxidative phosphorylation and ATP production. This energy deficit further compromises renal cell function, leading to cell injury and apoptosis (276, 277). In particular, the accumulation of ceramides impairs mitochondrial function by altering mitochondrial membrane structure, promoting oxidative stress, and enhancing the production of reactive oxygen species (ROS). This cycle of mitochondrial dysfunction and lipid accumulation accelerates kidney injury, especially in podocytes, which are critical for glomerular filtration (276, 277).

Animal models have been instrumental in understanding the role of lipotoxicity and mitochondrial dysfunction in DKD. Studies have demonstrated that high-fat diets induce changes in podocyte structure, particularly in mitochondria, contributing to kidney damage (276, 277). Animal models have also shown that interventions targeting lipid metabolism, such as inhibiting ceramide synthesis or promoting mitochondrial protection, can improve kidney outcomes (278). For example, inhibiting serine palmitoyltransferase (the enzyme responsible for ceramide synthesis) in animal models has been shown to reduce lipid accumulation, inflammation, and fibrosis, leading to improved renal function (276, 277, 279).

On the same lines, a recent study shows the critical role of mitochondrial dysfunction and oxidative stress in the development of DKD (280). It highlights how metabolic changes in diabetes, including reprogramming of lipid metabolism, affect TECs. The research demonstrates that mitochondrial oxidative damage is a primary factor in TEC injury and lipid peroxidation caused by lipid accumulation. Using SS31, a tetrapeptide that interacts with phospholipid cardiolipin located within inner mitochondrial membrane and protects the structure of mitochondrial cristae, the study shows that protecting mitochondria significantly reverses the decreased expression of key enzymes involved in FAO and limits oxidative damage markers in DKD (280–284). The study also reveals that high glucose levels drive altered lipid metabolism, leading to lipid droplet formation and sphingolipid imbalance (280). These findings underscore the nephroprotective potential of SS31 through its impact on metabolic pathways, offering insights into novel therapeutic strategies for treating DKD.

### Mitochondrial Reactive Oxygen Species

In addition, mitochondrial reactive oxygen species (mtROS) play roles in adaptive responses, known as mitochondrial hormesis, where low levels of ROS can enhance cellular resilience to stress. Conflicting results in DKD research, where some models show increased mtROS, whereas others do not stem from different detection methods, models, and disease stages. This evolving understanding of mtROS suggests a nuanced role in DKD, where precise modulation rather than elimination of mtROS could offer therapeutic benefits. Since Brownlee (285) linked hyperglycemia-induced mtROS as a central mechanism for diabetic microvascular complications, this model has dominated the field. However, recent findings have sparked debate regarding the exact source and role of ROS in DKD. Although ROS-induced damage is generally accepted to be elevated in DKD, variations in observed mtROS levels arise due to differing detection methods, experimental models, and disease stages. For example, increased mtROS was reported in db/db mouse kidneys using a mitochondrial redox-sensitive green, fluorescent probe, whereas reduced mitochondrial superoxide levels were observed in streptozotocin-injected and Ins2-Akita mice following systemic dihydroethidium (DHE) administration, suggesting ROS contributions from other compartments like the endoplasmic reticulum or enzymes such as NADPH oxidase (Nox) (286). Interestingly, studies activating AMPK to enhance mitochondrial biogenesis and OXPHOS increased mtROS but concurrently improved DKD outcomes, indicating that mtROS alone may not drive DKD pathogenesis. Moreover, animal models enable targeted genetic manipulation, allowing researchers to investigate specific mitochondrial pathways involved in DKD. For instance, studies using Drp1 knockout mice demonstrate that inhibiting mitochondrial fission can protect podocytes from damage and reduce proteinuria in diabetes. Similarly, overexpression of the PINK1/Parkin pathway in diabetic models has been shown to restore mitophagy, thereby preventing mitochondrial dysfunction and protecting tubular cells from injury (287). In addition, these models are invaluable for preclinical testing of therapeutic strategies aimed at mitigating mitochondrial dysfunction. Potential treatments, such as mitochondrial antioxidants, modulators of mitochondrial dynamics, and mitophagy activators, can be evaluated for their effectiveness in ameliorating DKD within animal systems before advancing to clinical trials.

## ROLE OF OXIDATIVE STRESS IN DKD

Renal oxidative stress is commonly a result of the upregulation of pro-oxidant enzymes, which induce the production of ROS, coupled with the depletion of antioxidants. ROS can damage cellular proteins, lipids, and DNA, leading to cellular dysfunction (288–292). Accumulating evidence has demonstrated that the overproduction of ROS serves as a key link between altered metabolic pathways in the kidneys and disrupted renal hemodynamics associated with diabetic nephropathy. These metabolic disturbances ultimately contribute to inflammation and fibrosis (293–297).

In biological systems, molecular oxygen undergoes a series of reductive biosynthetic steps, forming reactive intermediates that are oxygen-derived, unstable, highly reactive, and energetically small molecules. ROS include both free radical molecules, such as superoxide (O_2_^−^), hydroxyl (OH), peroxyl (ROO), and alkoxyl (RO), as well as nonradicals like hypochlorous acid (HOCl), ozone (O_3_), and hydrogen peroxide (H_2_O_2_). These nonradical ROS are oxidizing agents that can be easily converted into free radicals.

Mitochondria are the primary source of endogenous ROS, with up to 16 different sites of ROS production (298–300). Key contributors to mitochondrial ROS production include OXPHOS, respiratory chain uncoupling, and the dysregulation of complex I, coenzyme Q, and complex III (301).

In the context of hyperglycemia, glycolysis and the citric acid cycle generate increased amounts of reduced nicotinamide adenine dinucleotide (NADH). Enhanced NADH recycling by mitochondrial complex I leads to electron leakage, resulting in increased ROS production (302). The elevated levels of both NADH and ROS can inhibit and inactivate glyceraldehyde 3-phosphate dehydrogenase (GAPDH), thereby blocking the glycolytic pathway. This blockage causes the accumulation of glycerol 3-phosphate and its intermediates, which subsequently initiate alternative glucose metabolic pathways, such as the polyol and advanced glycation end product (AGE) pathways, further exacerbating cellular oxidative stress (302).

Superoxide anions, the primary ROS produced by mitochondria, are not diffusible due to their negative charge but are rapidly dismutated to H_2_O_2_ either by superoxide dismutase 2 (SOD2) in the mitochondrial matrix or SOD1 in the intermembrane space, depending on the site of generation. Unlike superoxide, H_2_O_2_ is diffusible and can move from the mitochondria into the cytosol, contributing to the overall cellular ROS pool, along with H_2_O_2_ generated by NADPH oxidase 4 (NOX4) (303).

Among ROS, H_2_O_2_ is the dominant cellular oxidant and plays a crucial role in redox regulation (304). It influences various cellular signaling pathways, including the activation of MAPK or PI3K, accumulation of HIF-1α in the nucleus, and NF-κB-mediated transcription of proinflammatory cytokines. The concentration of H_2_O_2_ in both extracellular and intracellular spaces is critical for cellular metabolism and survival, maintained through a balance between its formation and scavenging by enzymes. Defense systems against H_2_O_2_ include catalase (CAT), primarily found in peroxisomes; glutathione peroxidases (GPXs); and peroxiredoxins (PRXs). CAT facilitates the conversion of H_2_O_2_ into water (H_2_O) and molecular oxygen (O_2_). GPXs and PRXs, located in various cellular compartments, are integral to preventing H_2_O_2_ accumulation in specific regions. GPX, a selenium-containing enzyme, catalyzes the reduction of H_2_O_2_ and lipid peroxides into water and lipid alcohols (305). Both PRXs and GPXs undergo oxidation/reduction of cysteine or selenocysteine residues. For instance, when H_2_O_2_ interacts with an oxidized PRX, it can transfer the oxidation to target proteins like phosphatases or transcription factors (306). In addition, thioredoxin/thioredoxin reductase (Trx/TrxR) and glutathione (GSH)/glutathione reductase (GR) systems cooperate to maintain the reduced, active form of enzymes such as PRXs and GPXs, enabling the reversibility of redox modifications caused by H_2_O_2_ (307). The PRX family consists of six members with distinct amino acid sequences and at least one peroxidatic cysteine in the active site, and they are localized across multiple cellular compartments including the membrane, cytosol, mitochondria, endoplasmic reticulum, nucleus, and peroxisomes (306, 308).

After mitochondria, NADPH oxidases (Noxs) represent the second major source of ROS in the kidneys (309). Noxs are considered “professional ROS producers” due to their primary role in generating ROS. The first identified isozyme, gp91phox (now Nox2), was found in phagocytic cells. Subsequently, six additional homologues have been identified in nonphagocytic cells (Nox1, Nox3, Nox4, Nox5, Duox1, and Duox2) (310). Noxs are located not only in the plasma membrane (Nox1-5 and Duox1-2) but also in the endoplasmic reticulum (Nox2, Nox4, and Nox5), mitochondrial membrane (Nox4), and nuclear membrane (Nox4 and Nox5) (311). Noxs catalyze the electron transfer from NADPH to oxygen molecules, producing ROS such as superoxide anion and, in the case of Nox4, hydrogen peroxide (312).

Unlike other enzymes that produce ROS as a by-product of their activity, Noxs are unique in their ability to directly generate ROS (297). Under normal physiological conditions, these enzymes remain inactive. However, in high glycemic environments, they become activated, leading to increased transcription and translation.

In DKD, Nox4 is the dominant isoform expressed in the kidneys. Initially named “Renox” due to its high constitutive activity in the kidney, Nox4 is upregulated in diabetes and associated with increased ROS production and renal injury (313). Genetic manipulation or pharmacological inhibition of Nox4 has shown protective effects in diabetic nephropathy models, such as in STZ-induced *ApoE*(−/−) mice (313). Nox4’s role in podocyte injury in diabetes has been confirmed using podocyte-specific Nox4 knockout mouse models rendered diabetic via STZ injections (314).

Due to the complexity of ROS species, their direct detection remains a significant challenge for in vivo studies. To address this, redox-sensitive probes and oxidative stress biomarkers, such as AGEs, oxidized low-density lipoprotein (oxLDL), malondialdehyde (MDA), 8-hydroxy-2^′^-deoxyguanosine (8-OHdG), and advanced oxidation protein products (AOPPs), are commonly used as relatively unbiased tools for ROS detection (315, 316). Recent advancements have enabled dynamic monitoring of ROS in diabetic kidneys using reduction-oxidation-sensitive green fluorescent protein (roGFP) bio-sensors targeted to the mitochondrial matrix. This approach offers a more accurate assessment of mitochondrial ROS levels, minimizing biases associated with traditional superoxide detection methods (310).

Finally, lipid peroxidation represents a key downstream consequence of oxidative stress and a major mechanism of membrane damage. This process is a free radical-mediated process that damages cell membranes by oxidizing polyunsaturated fatty acids (PUFAs) in lipids. It proceeds through three main stages: initiation, propagation, and termination. ROS, such as hydroxyl radicals, can abstract hydrogen atoms from lipids, forming lipid radicals. These lipid radicals react with oxygen to form lipid peroxyl radicals, which then attack nearby lipids, creating a self-amplifying chain reaction. The reaction halts when radicals are neutralized by antioxidants or combine into stable products (317, 318).

The end products of lipid peroxidation include malondialdehyde (MDA) and 4-hydroxynonenal (4-HNE)—toxic molecules that are commonly used as biomarkers of oxidative stress (319, 320).

Lipid peroxidation is not only a marker of oxidative damage but also plays an active role in cell death pathways. Notably, it is a key driver of ferroptosis, an iron-dependent, nonapoptotic form of regulated cell death. Ferroptosis is characterized by the overwhelming accumulation of lipid peroxides, particularly in membrane phospholipids. It is distinct from other cell death types in morphology and biochemistry and is inhibited by lipid antioxidant enzymes like GPX4 (glutathione peroxidase 4).

Ferroptosis has been implicated in various diseases, including neurodegeneration, ischemia-reperfusion injury, and cancer, making lipid peroxidation not just a byproduct of damage, but a potential therapeutic target (321).

## ROLE OF INFLAMMATION IN DKD

Recent advances in research have highlighted the critical role of inflammation in the pathogenesis of DKD. Far from being a mere consequence of metabolic dysfunction, inflammation drives kidney injury by modulating immune responses, promoting oxidative stress, and exacerbating fibrotic processes. The inflammatory paradigm in DKD encompasses innate and adaptive immune pathways involving a broad spectrum of proinflammatory cytokines, chemokines, and immune cells. These mediators converge to amplify renal tissue damage, fueling the cycle of chronic inflammation and fibrosis that typifies DKD (322). Understanding the inflammatory mechanisms underlying DKD sheds light on the disease’s progression and opens new avenues for biomarker discovery and therapeutic intervention.

Current treatment options, including renin-angiotensin-aldosterone system inhibitors and sodium-glucose cotransporter-2 inhibitors, have shown promise in reducing disease progression, yet they fall short of halting it entirely. The development of therapies explicitly targeting inflammatory pathways offers a compelling strategy to address this unmet need. Indeed, preclinical and clinical studies have begun to unravel novel anti-inflammatory agents with the potential to attenuate renal damage and preserve kidney function in patients with DKD (323). As research advances, a more nuanced understanding of the interplay between inflammation and DKD pathogenesis may revolutionize therapeutic approaches and improve patient outcomes.

### Pathophysiology of Inflammation in DKD

#### Oxidative stress and its interplay with inflammation.

Oxidative stress, marked by an imbalance between ROS and antioxidant defenses, is a central player in DKD pathogenesis. In DKD, hyperglycemia drives excessive ROS generation through mechanisms such as mitochondrial dysfunction, activation of the polyol pathway, and AGE formation. This excessive ROS generation induces renal injury by promoting endothelial dysfunction, podocyte damage, and tubular apoptosis. ROS contributes directly to renal injury by damaging cellular structures, including DNA, proteins, and lipids (324, 325).

Oxidative stress also amplifies inflammation by activating key signaling pathways such as nuclear factor-κB (NF-κB) and mitogen-activated protein kinase (MAPK), which lead to the release of proinflammatory cytokines and chemokines (324, 325). For example, the peroxisome proliferator-activated receptor gamma coactivator-1 alpha (PGC-1α), a regulator of mitochondrial biogenesis and antioxidant defense, is often suppressed in DKD. Dysregulation of PGC-1α impairs mitochondrial function, enhancing ROS production and triggering inflammatory cascades (326). This suppression seen in DKD exacerbates oxidative stress and promotes inflammation, forming a vicious cycle wherein oxidative stress and inflammation mutually reinforce each other, driving DKD progression (326).

#### Immune cell activation in DKD.

The innate and adaptive immune systems are crucial in mediating inflammation in DKD. Hyperglycemia and oxidative stress act as danger signals, activating immune cells such as macrophages, dendritic cells, and lymphocytes. The activation and infiltration of immune cells in renal tissue represent a hallmark of DKD inflammation. Both innate and adaptive immune systems contribute to the disease, with macrophages, T cells, and dendritic cells playing pivotal roles (322, 327).

Macrophages are key players in DKD inflammation. They exhibit plasticity and can polarize into proinflammatory M1 or anti-inflammatory M2 phenotypes. DKD has an imbalance favoring M1 macrophages, which produce proinflammatory cytokines like tumor necrosis factor-alpha (TNF-α) and inter-leukin-1 beta (IL-1β). M1 macrophages produce proinflammatory cytokines that exacerbate renal damage, whereas M2 macrophages, though potentially reparative, may also promote fibrosis in chronic conditions. This polarization exacerbates tissue damage and fibrosis (328).

T-cell dysregulation, including an imbalance in T-helper subsets, further intensifies inflammation by secretion of interferons and interleukins (328). These immune responses sustain inflammation and contribute to the fibrotic remodeling characteristic of DKD. T cells, particularly CD4^+^ subsets, also contribute to DKD pathogenesis. Th1 and Th17 cells are upregulated in DKD and secrete proinflammatory cytokines, whereas regulatory T cells (Tregs), which suppress inflammation, are often reduced. This dysregulation further amplifies the inflammatory environment in the kidney (322, 327).

#### Cytokine and chemokine dynamics.

Inflammatory cytokines and chemokines are pivotal mediators of kidney damage in DKD. These molecules are produced by immune cells, resident renal cells, and endothelial cells in response to hyperglycemia and oxidative stress. Proinflammatory cytokines and chemokines orchestrate the inflammatory environment in DKD, mediating renal injury. Key cytokines, including TNF-α, IL-1β, and interleukin-6 (IL-6), drive inflammation by activating signaling pathways that promote fibrosis, endothelial dysfunction, and apoptosis. For instance, TNF-α induces podocyte injury and increases vascular permeability, contributing to albuminuria. IL-6 exacerbates renal fibrosis by stimulating collagen synthesis and myofibroblast activation (329). In addition, IL-1β and IL-6 exacerbate glomerular and tubular damage through leukocyte recruitment and activation (329, 330). Chemokines, including monocyte chemoattractant protein-1 (MCP-1), recruits monocytes and macrophages to the kidney, sustaining the inflammatory response and promoting tissue damage (330, 331). Elevated levels of these inflammatory mediators correlate with the severity of DKD, highlighting their potential as biomarkers and therapeutic targets (331).

#### Endoplasmic reticulum stress and inflammation.

Endoplasmic reticulum (ER) stress is increasingly recognized as a link between metabolic dysregulation and inflammation in DKD. ER stress arises from the accumulation of misfolded or unfolded proteins in the ER, a condition commonly observed in DKD due to hyperglycemia, lipid accumulation, and oxidative stress. Triggered by ER stress, the UPR initially aims to restore ER homeostasis; prolonged activation leads to proinflammatory signaling through pathways like PERK and IRE1α (332, 333). Chronic ER stress in DKD activates inflammatory signaling pathways such as the c-Jun N-terminal kinase (JNK) pathway and NF-κB, promoting the production of proinflammatory cytokines. ER stress also impairs autophagy, a cellular process for clearing damaged organelles and proteins. The inhibition of autophagy in DKD exacerbates inflammation and cellular injury in the diabetic kidney by failing to clear dysfunctional mitochondria and protein aggregates, further stimulating ROS production and cytokine release (333). The interplay between oxidative stress, immune cell activation, cytokine dynamics, and ER stress creates a self-sustaining cycle of inflammation in DKD. These mechanisms not only contribute to renal injury but also offer potential therapeutic targets to interrupt the progression of this debilitating condition. Understanding these pathways in greater detail may pave the way for novel anti-inflammatory interventions in DKD.

### Clinical Biomarkers of Inflammation in Diabetic Kidney Disease

#### Established biomarkers.

C-reactive protein (CRP), cytokines, and adhesion molecules are among the most extensively studied inflammatory biomarkers in DKD. CRP, an acute-phase reactant produced by hepatocytes, is elevated in response to systemic inflammation and correlates with the severity of renal dysfunction. Studies indicate that high serum CRP levels are predictive of rapid DKD progression and associated cardiovascular risks (334).

Cytokines, such as TNF-α, IL-6, and IL-1β, have well-documented roles in promoting renal inflammation and fibrosis. These proinflammatory mediators activate signaling pathways, such as NF-κB, that amplify inflammatory responses and contribute to endothelial dysfunction (335). Their elevated levels in serum and urine have been associated with worsening renal outcomes in patients with DKD.

Adhesion molecules, including intercellular adhesion molecule-1 (ICAM-1) and vascular cell adhesion molecule-1 (VCAM-1), facilitate leukocyte recruitment to sites of inflammation. Increased levels of these molecules indicate endothelial dysfunction and are linked to albuminuria and the progression of renal damage in patients with DKD (4). Urinary biomarkers, such as MCP-1, are gaining recognition as noninvasive markers of renal inflammation. MCP-1 reflects monocyte infiltration into the kidney and is significantly elevated in DKD, correlating with the degree of tubulointerstitial inflammation and fibrosis (324).

#### Emerging biomarkers.

Advances in molecular biology and proteomics have led to the discovery of novel biomarkers that provide deeper insights into the inflammatory mechanisms underlying DKD. Circulating inflammatory protein signatures, such as tumor necrosis factor receptors TNFR1 and TNFR2, have emerged as strong predictors of end-stage renal disease (ESRD) in diabetes. Elevated levels of these receptors indicate sustained TNF-α signaling, which exacerbates inflammation and tissue injury. Proteomic analyses have demonstrated that these biomarkers have significant prognostic value for identifying high-risk patients (336).

The stimulator of interferon genes (STING) pathway has gained attention as a key regulator of inflammation in DKD. Targeting the STING pathway offers a promising therapeutic strategy for modulating the inflammatory response in DKD (337). The STING pathway is a pivotal component of the innate immune system, primarily recognized for its role in detecting cytosolic DNA and initiating inflammatory responses. Activation of this pathway is intricately linked to the pathogenesis of diabetic kidney disease, where it contributes to renal inflammation and injury.

The STING pathway is activated by the cyclic GMP-AMP synthase (cGAS), which senses double-stranded DNA in the cytosol. Upon detection, cGAS synthesizes cyclic GMP-AMP (cGAMP), a second messenger that binds to and activates STING located on the endoplasmic reticulum membrane. Activated STING then translocates to the Golgi apparatus, facilitating the phosphorylation of TANK-binding kinase 1 (TBK1). TBK1 subsequently phosphorylates interferon regulatory factor 3 (IRF3), leading to its dimerization and nuclear translocation. In the nucleus, IRF3 induces the expression of type I interferons and other proinflammatory cytokines, amplifying the immune response.

In the context of DKD, mitochondrial dysfunction plays a crucial role in activating the cGAS-STING pathway. Hyperglycemia-induced oxidative stress leads to mitochondrial damage, resulting in the release of mitochondrial DNA into the cytosol. This mtDNA is recognized by cGAS, triggering the STING pathway and promoting inflammation. Studies have demonstrated that in diabetic conditions, such as in db/db mice, there is an upregulation of the cGAS-STING pathway in podocytes, the specialized cells essential for glomerular filtration (338). This activation is associated with increased phosphorylation of TBK1 and NF-κB, leading to podocyte injury and proteinuria (338). Furthermore, the nucleotide-binding oligomerization domain-like receptor family pyrin domain-containing 3 (NLRP3) inflammasome, another critical component of the innate immune system, has been implicated in DKD (339). Mitochondrial damage and the subsequent release of mtDNA can activate the NLRP3 inflammasome, leading to the production of proinflammatory cytokines such as IL-1β and IL-18 (339). This process exacerbates renal inflammation and fibrosis, contributing to the progression of DKD (339).

Recent research has highlighted the therapeutic potential of targeting the cGAS-STING pathway in DKD. Enhancing nicotinamide adenine dinucleotide (NAD^+^) metabolism through supplementation with NR has been shown to improve mitochondrial function, reduce mtDNA release, and subsequently attenuate cGAS-STING-mediated inflammation (265). In this study NR treatment of db/db mice resulted in decreased albuminuria and renal injury markers, effects attributed to the inhibition of the cGAS-STING pathway and reduced inflammatory responses (265). Another recent study also notes the activation of cGAS-STING signaling in diabetic kidneys, with increased expression of both cGAS and STING in diabetic db/db mice (340). This activation was associated with elevated phosphorylation of TBK1 and IRF3, confirming downstream pathway engagement (340). Importantly, mitochondrial dysfunction appears to be a key driver of cGAS-STING activation in DKD, as indicated by increased mitochondrial DNA damage and reduced complex I activity in db/db kidneys (340). The restoration of mitochondrial function by pharmacological interventions, such as sacubitril/valsartan (Sac/Val), effectively suppressed STING pathway activation, suggesting that mitochondrial integrity is essential for limiting inflammatory cascades in DKD (340). Although this study reported robust cGAS-STING activation, there was relatively minimal engagement of the absent in melanoma 2 (AIM2) inflammasome and Toll-like receptor 9 (TLR9) in KKAy mice. This suggests that self-DNA sensing via STING may be a dominant driver of inflammation in diabetic kidneys, particularly in models with pronounced mitochondrial dysfunction. Further downstream, STING activation has been linked to NF-κB signaling, a crucial transcriptional regulator of proinflammatory cytokines such as MCP-1 and TNF-α (340). Inhibition of STING in diabetic models not only reduced inflammatory cytokine expression but also mitigated renal fibrosis, indicating that STING-targeted therapies may hold promise for DKD treatment. In addition, STING appears to regulate the phosphorylation of signal transducer and activator of transcription 3 (STAT3), further amplifying inflammatory signaling (340).

The cGAS-STING pathway serves as a critical mediator of inflammation in DKD, primarily through the detection of cytosolic mtDNA resulting from mitochondrial dysfunction. Its interplay with other innate immune components, such as the NLRP3 inflammasome, underscores the complex network of inflammatory signaling contributing to renal injury. Therapeutic strategies aimed at modulating this pathway, including enhancing NAD^+^ metabolism, hold promise in mitigating inflammation and slowing the progression of DKD.

Insulin signaling pathways also play a critical role in the interplay between metabolic dysfunction and inflammation. Dysregulated insulin signaling contributes to chronic low-grade inflammation, perpetuating a cycle that worsens metabolic and renal outcomes. Understanding these pathways provides a foundation for developing targeted therapies to break this cycle (337).

Exploring clinical biomarkers in DKD has revealed both established markers, such as CRP and cytokines, and emerging candidates, such as TNFRs and the STING pathway, that offer enhanced predictive and therapeutic potential (Table 1). These biomarkers improve our understanding of DKD pathophysiology and pave the way for precision medicine approaches to mitigate the inflammatory burden in this condition.

### Therapeutic Targeting of Inflammation in Diabetic Kidney Disease

#### Anti-Inflammatory pharmacotherapies.

One of the most significant advances in treating DKD has been identifying anti-inflammatory effects in existing pharmacological agents. Sodium-glucose cotransporter-2 (SGLT2) inhibitors, initially developed for glycemic control, have demonstrated pronounced anti-inflammatory properties. SGLT2 inhibitors like canagliflozin attenuate renal fibrosis and endothelial dysfunction by reducing glucose-induced oxidative stress and modulating inflammatory mediators. Studies reveal a reduction in key inflammatory markers such as TNF-α and IL-6, suggesting their potential to interrupt the inflammatory cascade driving DKD progression (335).

Metformin, another cornerstone therapy for type 2 diabetes, exhibits anti-inflammatory effects through mechanisms involving AMP-activated protein kinase (AMPK) activation. By inhibiting NF-κB signaling, metformin reduces the production of proinflammatory cytokines, such as TNF-α and MCP-1. Preclinical studies have shown that metformin’s modulation of oxidative and inflammatory pathways significantly attenuates renal injury in diabetic models, highlighting its dual role in metabolic regulation and inflammation suppression (341).

#### Novel interventions and trials.

As understanding of the inflammatory landscape in DKD deepens, novel therapeutic interventions targeting specific cytokines and immune pathways are emerging. Cytokine-targeted therapies, such as monoclonal antibodies against IL-1β, such as canakinumab, have shown promise in clinical trials by reducing systemic inflammation and improving renal outcomes. Similarly, inhibitors of TNF-α signaling are being explored for their ability to mitigate renal fibrosis and immune cell recruitment. These approaches represent the forefront of precision medicine in DKD (323).

Immune modulation is another innovative strategy, with therapies targeting the Nrf2 pathway gaining traction. Nrf2, a key regulator of oxidative stress and inflammatory responses, protects against cellular injury by inducing antioxidant enzyme expression. Pharmacological activation of Nrf2 has been demonstrated to suppress inflammatory cytokine production and improve renal function in DKD models. Clinical trials of Nrf2 activators, such as bardoxolone methyl, have yielded encouraging results, suggesting that immune modulation may complement existing therapeutic regimens (342).

Although pharmacological interventions form the backbone of DKD management, lifestyle modifications are pivotal in mitigating inflammation. Dietary patterns rich in anti-inflammatory nutrients, such as omega-3 fatty acids, fruits, and vegetables, have been associated with lower levels of CRP and IL-6. In addition, caloric restriction and weight management reduce adipose tissue-derived proinflammatory cytokines, thereby alleviating systemic and renal inflammation (343). Regular physical activity offers complementary benefits by improving insulin sensitivity and reducing oxidative stress, both of which are key contributors to the inflammatory environment in DKD. Furthermore, emerging evidence supports the role of nutraceuticals, such as curcumin and resveratrol, in attenuating inflammation through antioxidant and anti-inflammatory mechanisms. Although promising, these approaches require validation in large-scale clinical trials to establish efficacy and safety profiles (324, 344).

#### Limitations in current biomarkers and therapeutic strategies.

Although biomarkers such as CRP, cytokines, and urinary albumin have advanced our ability to monitor DKD progression, they lack specificity and sensitivity in capturing the complexity of inflammatory processes in DKD. For instance, CRP is a generalized marker of systemic inflammation that does not distinguish between the myriad pathways contributing to kidney injury (334). Similarly, cytokines, such as IL-6 and TNF-α, provide valuable insights into inflammation but are influenced by comorbidities like cardiovascular disease and obesity.

Therapeutic strategies targeting inflammation in DKD face challenges in translating preclinical findings into clinical success. Current anti-inflammatory agents, including SGLT2 inhibitors and metformin, show promise in modulating inflammation, but their mechanisms are incompletely understood, and their efficacy may vary among individuals (335, 341). Moreover, these treatments are often prescribed alongside standard care, making isolating their specific anti-inflammatory effects difficult.

#### Need for personalized medicine approaches.

The heterogeneous nature of DKD underscores the need for personalized medicine. Interindividual variations in genetic, metabolic, and immune profiles influence the inflammatory pathways driving disease progression. For example, polymorphisms in genes encoding cytokines and adhesion molecules may alter inflammatory responses, impacting treatment outcomes (4). Despite these insights, current clinical practice predominantly relies on a one-size-fits-all approach, which may not adequately address the diverse needs of all patients with DKD.

Integrating precision medicine into DKD management requires developing robust patient stratification tools. These tools could leverage molecular profiling to identify subgroups most likely to benefit from specific therapies. Advances in computational biology and artificial intelligence offer opportunities to analyze complex datasets, enabling the identification of novel biomarkers and therapeutic targets tailored to individual patients.

#### Potential for integrating multiomics data in DKD research.

The emergence of multiomics technologies, including genomics, transcriptomics, proteomics, and metabolomics, could revolutionize DKD research. These approaches enable a comprehensive characterization of molecular pathways underlying inflammation and kidney injury, offering insights beyond those provided by single biomarkers (5). For example, proteomic studies have identified circulating protein signatures predictive of end-stage renal disease, highlighting the utility of such data in prognostication.

Integrating multiomics data with clinical information could facilitate the discovery of interconnected pathways driving DKD progression. Furthermore, these approaches may uncover novel therapeutic targets, such as pathways involved in immune modulation or oxidative stress, which are underexplored in current treatments. However, challenges such as data standardization, integration, and interpretation remain significant barriers to the widespread adoption of multiomics in clinical practice.

## ROLE OF CELL INJURY IN DKD

### Ferroptosis

Mechanisms of ferroptosis: Ferroptosis is a nonapoptotic, iron-dependent form of regulated cell death characterized by iron-dependent accumulation of lipid peroxides. Iron ions drive the Fenton reaction, catalyzing the formation of iron radicals, which cause lipid peroxidation in polyunsaturated fatty acids (PUFAs) in membrane phospholipids (345). Lipid peroxidation can also be driven enzymatically through lipoxygenases, which form hydroperoxides from PUFAs (345). The accumulation of these phospholipid peroxides leads to cell membrane rupture, the key moment that causes cell death by ferroptosis (345). Ferroptosis is primarily regulated by glutathione peroxidase 4 (GPX4), which protects against ferroptosis by reducing lipid hydroperoxides to their corresponding alcohols using glutathione (GSH) as a cofactor (346). This prevents the buildup of lipid peroxides, thereby preventing ferroptosis. Since GPX4 requires GSH to function properly, ferroptosis is also regulated by System Xc, which regulates cysteine uptake and GSH function (346).

Ferroptosis and DKD: Hyperglycemic and hyperlipidemic environments in diabetic kidney disease (DKD) lead to the overproduction of reactive oxygen species, which can react with iron to cause lipid peroxidation through the Fenton reaction. Increased iron uptake is caused by degradation in hepcidin and ferroportin proteins, which regulate iron trafficking. Studies have shown that lower GPX4 mRNA levels in the kidney biopsy of diabetic patients versus nondiabetic patients (347). In addition, GPX4 level is positively correlated with estimated glomerular filtration rate (eGFR) levels and negatively correlated with proteinuria, a hallmark of DKD (347). Furthermore, SLC7A11, a subunit of the system Xc complex, was significantly downregulated in the kidney biopsy of patients with diabetes (348). NRF2, a major regulator of ferroptosis due to its antioxidant functions and regulation of iron metabolism, was found to be decreased in DKD models (349). Knockdown of NRF2 increased sensitivity to ferroptosis in diabetic kidney disease, and upregulation rescued ferroptosis in high glucose HK-2 cells (350). Known inhibitors of ferroptosis are being experimented with as possible treatments for DKD. Studies have shown that Fer-1, which inhibits ferroptosis by expressing antioxidant genes, alleviates cell death and TGFB2-induced ferroptosis in the diabetic kidney (350).

### Necroptosis

Mechanisms of necroptosis: Necroptosis is a nonapoptotic programmed form of inflammatory cell death. A distinctive feature of necroptosis is the absence of activated caspases, like caspase 3/7/8, which are required for apoptosis or pyroptosis. Necroptosis is primarily induced by the cytokine TNFα, which stimulates its receptor TNFR1. TNFR1 signaling induces activation of RIPK1, which recruits RIPK3 to form the necroptosome (351, 352). A key characteristic of necroptosis is the absence of caspase 8, which is activated by TNFα and would lead to cell death by apoptosis (353). Phosphorylation of mixed lineage kinase domain-like pseudokinase (MLKL) by the necroptosome activates MLKL to insert itself into the cell membrane, inducing permeabilization and the inflammatory phenotype, as well as the release of damage-associated molecular patterns (DAMPs) (354, 355).

Necroptosis in DKD: Increased inflammation in DKD due to oxidative stress, hyperglycemia, and hyperlipidemia activates necroptosis pathways, which promote renal injury and fibrosis (356). Studies have shown that TNFα levels are increased in the serum and urine of patients with DKD (357). In addition, glomeruli of diabetic mice showed an increased level of phosphorylated RIPK1 (p-RIPK1) (356). RIPK3 is also heavily involved in the renal injury of diabetic mice, as RIPK3 inhibition reduced inflammation, attenuated kidney fibrosis, and increased the survival rate of mice with DKD (358). Diabetic mice also showed increased expression of phosphorylated MLKL (p-MLKL) (356). Currently, RIPK1/3 inhibitors are being experimented with as possible inhibitors of necroptosis in DKD. Nec-1, a known RIPK1/3 inhibitor, decreased hyperglycemia-induced necroptosis and inflammation (356).

### NETosis

Mechanisms of NETosis: NETosis is a form of programmed cell death characterized by the formation of neutrophil extracellular traps (NET), release of granule components into the cytosol, and decondensation of chromatin (359). At the final stage of NETosis, pores are formed in the plasma membrane, and both chromatin and NETs are released into the environment. Pores are created by the activation of gasdermin D (GSDMD), similar to pyroptosis (359). However, in contrast to pyroptosis, GSDMD is cleaved by neutrophil elastase. NETosis can be induced by a variety of factors, including ROS from NADPH oxidase and mitochondrial signaling (359).

NETosis and DKD: NETosis is induced in DKD due to elevated ROS and mitochondrial signaling. Studies have shown that DKD features an increase in the expression of NETosis markers, including citrullinated H3 and Myeloperoxidase (MPO), which are required for chromatin decondensation and NET formation (360). A greater number of neutrophils are primed toward NETosis in diabetic kidney disease as well (360). In addition, inhibition of protein arginine deiminase 4 (PAD4), which is required for histone modification in NETosis, significantly ameliorated renal injury in DKD mice (361). Thus, NETosis is being currently looked at as a possible therapeutic target for DKD.

### Apoptosis

Mechanisms of apoptosis: apoptosis is a form of caspase-dependent programmed cell death. It is characterized by two main pathways, the intrinsic and the extrinsic. The intrinsic pathway is characterized by internal signals, such as oxidative stress and DNA damage, which lead to the release of proapoptotic factors by the mitochondria, such as cytochrome c (362, 363). The BCL-2 family, which comprises both proapoptotic and antiapoptotic proteins, form complexes in the mitochondrial membrane, which affect the release of these proapoptotic factors (364). Mitochondrial signaling leads to the activation of initiator caspases, namely, caspase 9 (365). This leads to the activation of execution caspases, like caspase 3 and 7, which lead to cell fragmentation and death (365).

The extrinsic pathway is characterized by external signals, mainly through the binding of death receptors to their ligands, namely, TNFR and CD95 (366, 367). Death receptor signaling leads to the activation of inducer proteins like tumor necrosis factor-α receptor-associated death domain and TNF receptor-associated factor 2 (TRAF2), which activate Fas-associated death domain protein (FADD) (367). FADD activates caspase 8, which leads to the activation of executioner caspases 3 and 7, which induce cell death (367).

Apoptosis and DKD: Apoptosis is a significant contributor to podocyte injury, tubular injury, and eventually fibrosis in DKD. Hyperglycemia and metabolic dysfunction cause dysfunctional mitochondrial signaling, often leading to the release of proapoptotic factors. In addition, high ROS levels due to high glucose levels can trigger the intrinsic apoptosis pathway, leading to the cell death of podocytes and renal tubular cells (368). Intrinsic apoptosis in DKD is highly regulated by the BCL2 family of proteins. Studies have shown that the expression of BCL-2 is decreased in biopsy of renal tissue of patients with DKD, and increasing BCL2 expression decreases cleaved caspase 3 activity (369). In addition, Bax (Bcl-2-associated X protein), a proapoptotic protein of the BCL2 family, is increased in the renal tissue of type 1 diabetic mice (369). Inhibition of BAX improved the histopathology of diabetic kidney tissue (369).

The extrinsic pathway is significant in DKD due to the systemic inflammation from hyperglycemic environments. Inflammatory cytokines such as TNFα have been found to be increased in the serum of type 1 diabetic kidneys and advanced renal failure (357). In addition, TNF receptors 1 and 2 have been found to be associated with end-stage renal disease (ESRD) in patients with type 2 diabetes (336). Inhibitors of TNF receptors are being investigated as possible treatments for DKD.

### Pyroptosis

Mechanisms of pyroptosis: Pyroptosis is a nonapoptotic form of cell death typically triggered by inflammatory signals and is characterized by the formation of gasdermin pores in the cell membrane, leading to cell swelling and eventual lysis (370, 371). It is induced by the formation of the inflammasome, which recognizes pathogen-associated molecular patterns (PAMPs) and DAMPs via cytosolic pattern recognition receptors (PRRs) (370). Inflammasome signaling leads to the release of proinflammatory cytokines, primarily IL-1β and IL-18, as well as caspase-1 (372). Caspase-1 cleaves GSDMD, which leads to the formation of pores in the cell membrane (372).

In the context of DKD, pyroptosis plays a significant role in the progression of kidney damage. Studies have shown that both caspase 11 and GSDMD mediate pyroptosis in mouse models of DKD. Expression levels of caspase 11 and cleaved GSDMD are elevated in podocytes of diabetic mice and are accompanied by increased inflammatory cytokines IL1b and IL18 (373). These changes were absent in a caspase-11 or GSDMD knockout model (373). Other gasdermin proteins, such as gasdermin E (GSDME), have been shown to mediate pyroptosis in diabetic kidney disease as well. Protein levels of GSDME were significantly elevated in the renal cortex of diabetic mice, and cleaved GSDME levels were found to be elevated 20 wk after STZ injection (374). In addition, both patient and murine models of DKD featured increased expression of NLRP3 and caspase-1 (375). Podocyte-specific caspase-1-deficient mice showed partial protection against DKD and podocyte-specific NLRP3 deficiency was fully protected against DKD (376). This suggests that pyroptosis plays a significant role in the progression of DKD and can be targeted for treatment.

### PANoptosis

PANoptosis is a recently defined form of programmed cell death, which combines features of pyroptosis (P), apoptosis (A), and necroptosis (N). It involves the cross talk and activation of several cell death molecules and is regulated through a multimeric protein complex named the PANoptosome. The PANoptosome has been shown to contain several cell death molecules, including RIPK1, NLRP3, and caspase 8 (377). However, other studies have shown that RIPK3, caspase 1, and caspase 6 are also components of the PANoptosome (378). Thus, the PANoptosome features death molecules that are critical to the mechanisms of pyroptosis, apoptosis, and necroptosis. PANoptosis can be induced by several factors, including inflammation and mitochondrial ROS (379, 380).

PANoptosis is currently being studied as a possible therapeutic target for DKD. Inhibiting PANoptosis has already been shown to ameliorate the pathology of diabetic retinopathy (381). In addition, a recent study has shown NAD + depletion as an inducer of PANoptosis via the immune sensor NLRC5 (382). Both NAD + metabolism and NLRC5 have been closely tied as significant regulators in the pathogenesis of DKD (265, 383). NLRC5 knockout (KO) mice exhibited lower albuminuria and macrophage infiltration, with higher levels of podocin and nephrin (383). Increased NAD metabolism through the supplement NR was found to decrease inflammation, albuminuria, and improve mitochondrial function (265). Thus, PANoptosis is an exciting new therapeutic target in the field of DKD research.

### Cuproptosis

Cuproptosis is another recently characterized form of copper-dependent programmed cell death (384). It is induced by the accumulation of copper in the mitochondria and its binding to lipoylated enzymes in the TCA cycle (385). The aggregation of these lipoylated proteins leads to the loss of Fe-S cluster-containing proteins, which subsequently induces cell death (385). Thus, cuproptosis is highly regulated by ferredoxin 1 (FDX1), which is an upstream regulator of protein lipoylation. *FDX1* KO was found to inhibit protein lipoylation and cuproptosis in vivo (385).

Cuproptosis is being studied as a possible mechanism of pathogenesis in DKD. Elevated Cu levels have been found in diabetic rat kidney models (386). The selective Cu chelator triethylenetetramine (TETA) was found to ameliorate renal injury and fibrosis and reduce TGFβ levels in the rat model of DKD (387). Thus, cuproptosis represents a possible target in the treatment of DKD.

In DKD, multiple forms of regulated cell injury and death play pivotal roles in driving renal dysfunction, inflammation, and fibrosis. Beyond the traditionally studied apoptosis, emerging mechanisms such as ferroptosis, necroptosis, NETosis, pyroptosis, PANoptosis, and cuproptosis have been increasingly implicated in DKD pathogenesis. Each cell death mechanism involves unique molecular triggers and signaling cascades, offering novel therapeutic targets (Table 2). Ongoing research into their modulation holds promise for the development of multitargeted interventions to halt or reverse DKD progression.

## ROLE OF EPIGENETIC MODIFICATIONS IN DKD

Kidney diseases are associated with various molecular changes that occur in renal cells, as well as in their environment. Numerous genetic alterations have been established and described in the course of renal diseases, including chronic kidney disease, kidney injury, renal fibrosis, and diabetic kidney disease. Impressive lists of genes involved in the development of diabetic kidney disease have been summarized in review articles (388, 389). Identification and analysis, e.g., Genome-wide Association Studies (GWAS) and exome sequencing, of these genetic traits are of utmost importance in for diagnosis and in discovery of effective therapies for renal diseases (390–392), including DKD (393–395). Importantly, most of these studies should still be confirmed in further experimental tests and analyses.

Nonetheless, genetic modifications alone do not explain all the changes in kidney function in physiology and in the course of renal diseases (396, 397). Currently, there is mounting evidence showing that not only genetic but epigenetic modifications play a role in the development of various kidney abnormalities, as demonstrated in research articles and summarized in recent excellent reviews (396, 398–401). Both the genetic and epigenetic analyses are necessary to fully understand the pathophysiology of DKD as well (402, 403).

Epigenetic modifications are defined as heritable changes in patterns of expression of the genes, leading to alterations of the phenotype, but without direct modifications to the DNA sequence (404, 405). These changes are dynamic, cell-specific, crucial to the understanding how particular cells respond to environmental cues, and they influence the development and progression of numerous diseases, kidney diseases included (406, 407). Important epigenetic modifications’ mechanisms playing a role in kidney diseases include: DNA methylation catalyzed by DNA methyltransferases; histone alterations (methylation, acetylation, phosphorylation, ubiquitination, butyrylation); and micro noncoding and long noncoding RNA (miRNA and lncRNA) expression (408–411).

### Metabolic Memory

Epigenetic changes were proposed as a mechanism explaining the phenomenon called “metabolic memory,” “hyperglycemic memory,” or “legacy effect” (412–415). It has been observed that hyperglycemia in patients, which is associated with critical issues related to diabetes, can lead to detrimental health complications, even after the glucose levels are normalized and the glucose control established (416–418). Through numerous clinical trials and modeling analyses, it has been proposed that the epigenetic changes (such as DNA methylation and histone acetylation) are responsible for the phenomenon of metabolic memory (419, 420). Some epigenetic mechanisms involved in metabolic memory were investigated, for example, histone H3 lysine 3 methylation and H3 lysine 9 demethylation at the NF-κB promoter in endothelial cells (415, 421, 422) or histone H3 lysine 9 trimethylation in inflammatory genes of vascular smooth muscle cells (423). These analyses are of the utmost importance, since metabolic memory can significantly impair patients’ health, as well as the effectiveness of potential therapies and diagnostic approaches (424–427).

### DNA Methylation

DNA methylation is one of the most common epigenetic mechanisms associated with DKD and occurs in CpG islands, where the methyl group is attached to the cytosine residue at 5’ position. The methylation reaction is conducted by DNA methyltransferases 1 and 3 (DNMT1 and DNMT3) (428, 429). Meanwhile, demethylation is caused by oxidation of methylcytosine and its conversion to 5-hydroxymethylcytosine. These processes can affect chromosome stability, structure, and interactions between DNA and proteins. DNA methylation can regulate gene expression in both directions, leading to either reduced (if occurs in promoter region) and enhanced (if occurs in gene body) expressions of the genes, which can promote the development of various diseases, including kidney disease (399, 401, 430, 431).

Similarly to GWAS studies, Epigenome-wide Association Studies (EWAS), performed mostly in blood-derived DNA, were done to evaluate epigenetic modifications that play roles in diabetic kidney disease (388, 432, 433). For example, one study identified numerous hypermethylated and hypomethylated genes (e.g., solute carrier family 22 member 12, transient receptor potential cation channel subfamily M member 6, aquaporin 9, and hyaluronidase 2; involved in kidney damage, injury, and kidney disease), associated with glucose metabolism, oxidative stress, and fatty acid metabolism (434). Methylation alterations in genes related to mitochondrial function (peptidase, mitochondrial processing subunit beta, Ts translation elongation factor, mitochondrial, and AU RNA binding methylglutaconyl-CoA hydratase) were shown as well to be related to kidney disease in patients with type 1 diabetes (435). Numerous DNA methylation markers associated with estimated glomerular filtration rate and subsequent decline in kidney function were described, too (436, 437). Extensive studies identified important genes that were methylated differently in individuals with type 1 diabetes and kidney disease versus individuals with diabetes but without evidence of kidney disease. These genes included: FKBP prolyl isomerase 5 (*FKBP5*) (its hypermethylation is associated with chronic kidney disease and type 2 diabetes), RUNX family transcription factor 3 (plays a role in TGF-β signaling pathway), *HDACs* (histone deacetylases involved in the development of kidney fibrosis), integrin subunit alpha L, and Pim-1 proto-oncogene, serine/threonine kinase (previously described as linked to renal cell carcinoma and breast cancer, respectively), PBX homeobox 1 (gene that regulates glucose metabolism), and cut like homeobox 1 (involved in kidney development and reported in chronic kidney disease) (438). Methylation of *FKBP5* and polypyrimidine tract binding protein 3, as well as solute carrier family 27 member 3 gene (which encodes a protein involved in lipid metabolism), was also emphasized as associated with DKD in the largest meta-analysis of DKD in type 1 diabetes to date (439).

Because epigenetic changes are cell-specific, DNA methylation alterations detected in blood samples may not correlate with those detected in other tissues, e.g., in renal tissue. Thus, it is crucial to use different tissue types for the analyses, even though obtaining a sufficient amount of DNA may be difficult in some of them (440). Such important analyses were performed in human kidney tubules and demonstrated DNA methylation changes in genes involved in kidney fibrosis (e.g., interferon gamma inducible protein 16) (441). Also, DNA hypomethylation of Syk (spleen tyrosine kinase), observed in kidney tissues, was associated with oxidative stress and apoptosis in diabetic kidney disease (442).

So far, a number of CpG sites with modified DNA methylation were identified; however, the mechanism and causality of these alterations are not clear. Importantly, besides the analyses done in various tissues, it is critical to gather more data from various ethnic groups (443, 444), especially if the data is to be used for the prediction of the potential therapeutic approaches for DKD (389, 445).

### Histone Modifications

Methylation and acetylation, as well as other histone modifications, regulate the DNA availability for transcription and, subsequently, can enhance gene expression. They are carried out by specific enzymes: methyltransferases, acetyltransferases, and histone deacetylases (HDACs), among others. HDACs play a role in fibrosis, inflammation, and injury of podocytes and tubules—so it was implicated that they are crucial for the DKD development, in both animal studies and in patients’ tissues (446–450). Importantly, these observations open a way to potential epigenetic therapies for patients with DKD. For example, sirtuins (NAD + dependent histone deacetylases) such as sirtuin-1 and sirtuin-3 decrease lipotoxicity- and high-glucose-mediated oxidative stress, inflammation, fibrosis, and apoptosis (451–456) in preclinical studies. Also, HDAC inhibitors have been already used in therapy of various diseases, including valproate for epilepsy and vorinostat for T-cell lymphoma. These drugs were also shown to alleviate some syndromes related to DKD, including renal tubular damage, prevent fibrosis, and decrease oxidative stress in diabetic mice and rats (449, 457–461). It has to be emphasized, however, that even though HDAC inhibitors are very promising potential agents in therapeutic approaches for numerous diseases—which includes their potential clinical benefit for the treatment of diabetic kidney disease—they are also associated with many adverse effects observed in patients (462–464).

Besides acetylation, histone methylation plays a very important role in DKD—changes in histone 3 lysine 4, lysine 9, and lysine 27 are associated with kidney injury mechanisms that occur in many renal cell types (podocytes, tubular cells, glomerular endothelial cells, and mesangial cells) (424, 465, 466). For example, histone-lysine N-methyltransferase enzyme EZH2 (enhancer of zeste homolog 2) is an enzyme that catalyzes the methylation of histone H3 at lysine 27. It regulates ferroptosis in tubular epithelial cells, which contribute to the DKD pathogenesis (467). Inhibiting EZH2 and subsequently decreasing methylation in H3K27 can lead to alleviation of podocyte dedifferentiation and podocyte damage (468); thus, EZH2 is considered a promising target for the treatment of various kidney diseases (469). Other inhibitors can mitigate renal fibrosis and inflammation (468, 470, 471) and protect podocytes as well (472). Moreover, losartan, an angiotensin II receptor blocker and a commonly used antihypertensive drug, reverses some H3 methylation epigenetic modifications and can act as a renoprotective factor (473).

DKD can be affected by other histone modifications, i.e., phosphorylation and ubiquitination (474–477) as well as butyrylation, a relatively novel epigenetic histone change induced by butyrate. Butyrate, a short-chain fatty acid, reduce renal inflammation and fibrosis in the course of the DKD, partially via histone lysine butyrylation in streptozotocin-induced DKD mouse model and in mesangial glomerular cells in vitro (478).

Importantly, various histone modifications (and various epigenetic alterations in general) may be involved together in regulation of the same mechanisms that play a role in DKD, for example, the activity of glucocorticoid receptor (479). Different epigenetic mechanisms (e.g., histone methylation and ubiquitination in this case) can be cross regulated as well (474).

### Non-Coding RNA Modifications

Long noncoding RNA H19 (LncRNA H19), the first maternally expressed gene discovered in eukaryotes, have been shown to possess a crucial role in diabetic kidney disease by regulating inflammation, fibrosis, mitochondrial function, apoptosis, and pyroptosis (480). Furthermore, LncRNA LINC00968 inhibits p21 expression via EZH2 and increases fibrosis in mesangial cells (481), and LncRNA *Tug 1* has been shown to be involved in mitochondria functioning in podocytes (482). Moreover, overexpression of micro-RNA 503 (miR-503) results in podocyte injury in a mouse model (483) and expression of miR-192, the most abundant micro-RNA in the kidney, was negatively correlated with urine albumin-creatinine ratio and with two markers of kidney fibrosis, fibronectin, and TGF-β1 (484). Interestingly, lncRNA can also act as regulators of miRNA in the course of kidney diseases (485). These examples demonstrate an important role the noncoding RNA plays in the pathogenesis of DKD and emphasize the need to explore that area in search for effective therapies for numerous renal diseases, including diabetic kidney disease (486–490). In general, the analyses of the epigenetic modifications in DKD, including the growing lists of potential therapeutics, are promising. However, many of these analyses were performed in preclinical models (in vitro and in vivo). It is crucial then to perform similar studies in human tissues and in clinical settings.

## CONCLUSIONS

**Figure 5.**
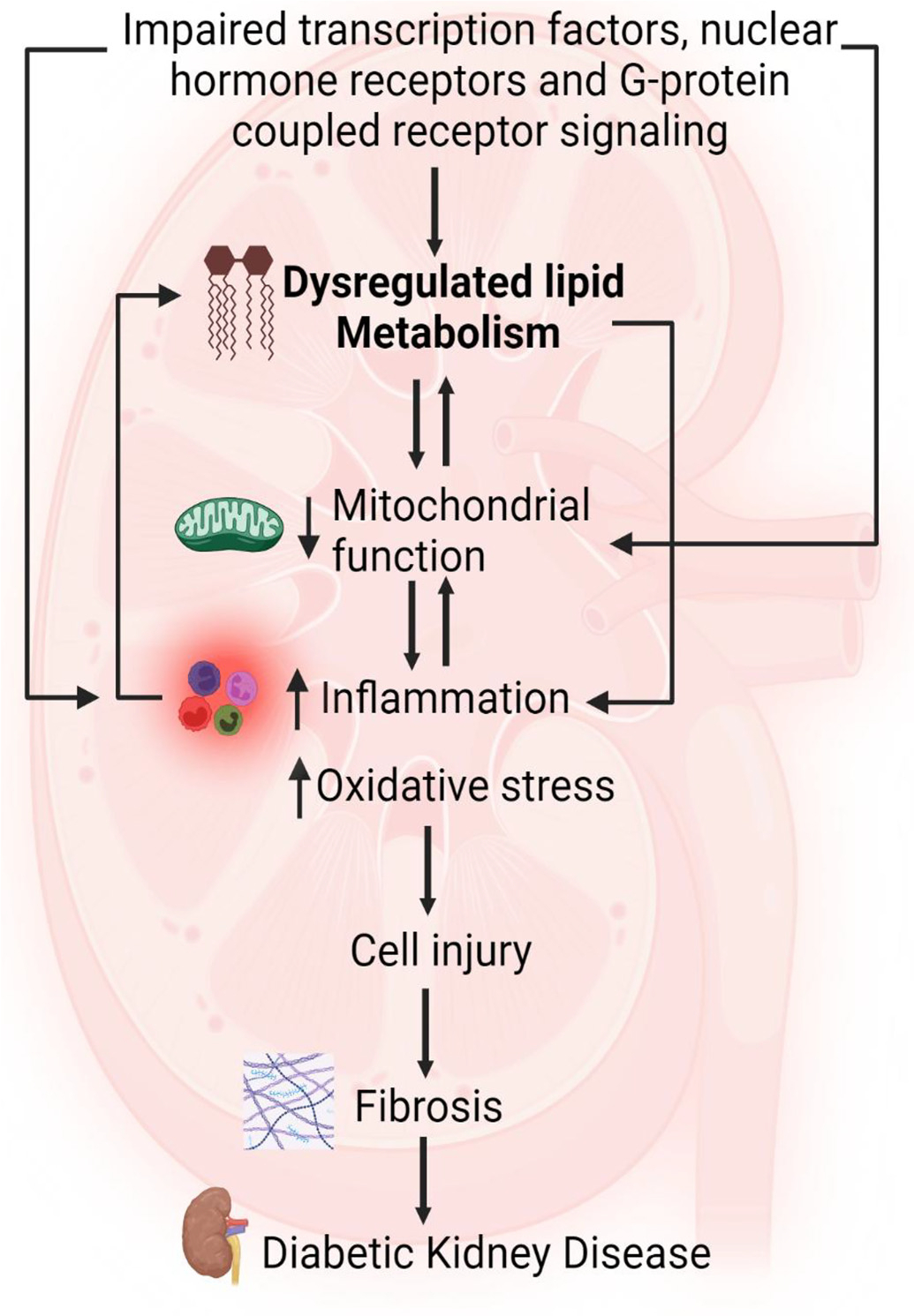
Summary of the interactions between nuclear hormone receptors, transcription factors, and G protein-coupled receptors in regulating lipid metabolism, mitochondrial function, oxidative stress, and inflammation that result in cell injury/cell death, fibrosis, and diabetic kidney disease.

Nuclear hormone receptors, transcription factors, and G protein-coupled receptors (GPCR) play interconnected roles in regulating lipid metabolism, mitochondrial function, oxidative stress, and inflammation, contributing to cell injury, fibrosis, and diabetic kidney disease (Fig. 5). This persistent inflammation and oxidative damage result in cellular injury, death, and activation of fibrotic pathways, contributing to extracellular matrix accumulation and renal fibrosis. Collectively, these molecular interactions drive the progression of DKD, leading to loss of kidney function.

Research in animal models highlights the potential for targeting lipid metabolism and mitochondrial function as therapeutic strategies for slowing or halting DKD progression (Table 3). Continued investigation into these mechanisms and how their modulation could reduce lipid accumulation, improve mitochondrial function, and alleviate inflammation and fibrosis in the kidneys could lead to novel and improved treatments for DKD and other kidney diseases. Future research should continue to explore new lines of molecular therapies that might offer better and longer-lasting renoprotective benefits than existing treatment strategies in patients with diabetes.

## Figures and Tables

**Table 1.** Summary of inflammation pathways

Activators	Description	References for Studies in DKD
TNFα	TNF-α induces podocyte injury and increases vascular permeability, contributing to albuminuria	(329, 335)
IL-1β	exacerbates renal fibrosis by stimulating collagen synthesis and myofibroblast activation	(329, 330, 335)
IL-6	exacerbate glomerular and tubular damage through leukocyte recruitment and activation	(329, 330, 335)
MCP1	Recruits monocytes and macrophages to the kidney, sustaining the inflammatory response and promoting tissue damage	(330, 331)
cGAS-STING	Detecting cytosolic DNA and initiating inflammatory responses	(265, 338)
NLRP3	Mitochondrial damage and the subsequent release of mtDNA can activate the NLRP3 inflammasome, leading to the production of proinflammatory cytokines	(339)
ICAM/VCAM	Facilitate leukocyte recruitment to sites of inflammation. Increased levels of these molecules indicate endothelial dysfunction and are linked to albuminuria and the progression of renal damage	(4)
CD4^+^ T cells	Th1 and Th17 cells are upregulated in DKD and secrete proinflammatory cytokines	(322, 327)

DKD, diabetic kidney disease; ICAM, intercellular adhesion molecule-1; IL-1β, interleukin-1 beta; IL-6, interleukin-6; MCP-1, monocyte chemoattractant protein-1; NLRP3, nucleotide-binding oligomerization domain-like receptor family pyrin domain-containing 3; TNFα, tumor necrosis factor-α; VCAM, vascular cell adhesion molecule.

**Table 2.** Summary of cell injury/death mechanisms

Mechanism	Description	References for Studies in DKD
Ferroptosis	Iron-dependent accumulation of lipid peroxides leads to cell membrane rupture.	(347–350)
Necroptosis	Nonapoptotic caspase-independent form of programmed inflammatory cell death.	(356–358)
NETosis	GSDMD activation drives the formation of pores in plasma membrane that release chromatin and neutrophil extracellular traps (NETs). GSDMD is cleaved by neutrophil elastase.	(360, 361)
Apoptosis	Caspase-dependent programmed cell death. Intrinsic pathway characterized by internal signal, such as oxidative stress and DNA damage. Extrinsic pathway by external signals, mainly binding of death receptors to their ligands (TNFR and CD95).	(336, 357, 368, 369)
Pyroptosis	Characterized by the formation of gasdermin pores in cell membrane, leading to cell swelling and eventual lysis. Caspase-1 cleaves GSDMD to trigger pore formation.	(373–376)
PANoptosis	Recently defined type of programmed cell death that combines features of pyroptosis (P), apoptosis (A), and necroptosis (N). Cross talk between several cell death molecules and regulated by PANoptosome protein complex, which contains several proteins including RIPK1, caspase 1, and caspase 3.	(381, 383)
Cuproptosis	Cell death is induced by accumulated mitochondrial copper bonding to lipoylated enzymes in the TCA cycle.	(386, 387)

GSDMD, gasdermin D; TCA, tricarboxylic acid; TNFR, tumor necrosis factor receptor.

**Table 3.** Summary of potential drug targets and biomarkers discussed in this review

Gene/Protein	Pathway	Note	References
FXR	Nuclear receptor, fatty acid	Therapeutic target	(80, 115, 116, 119, 120, 121)
TGR5	GPCR	Therapeutic target	(126–128)
VDR	Nuclear receptor	Therapeutic target	(81, 155, 160, 161)
ERRα	Nuclear receptor, mitochondria	Therapeutic target	(149, 151)
ERRγ	Nuclear receptor	Therapeutic target	(148, 150)
PPARα	Nuclear receptor, fatty acid, mitochondria	Therapeutic target	(89, 90, 102, 103, 105)
LXRα/β	Nuclear receptor, fatty acid, cholesterol	Therapeutic target	(167–169)
MR	Nuclear receptor	Therapeutic target	(176, 177, 180, 181)
SREBP-1	Transcription factor, fatty acid	Therapeutic target	(71, 74, 75, 79)
SREBP-2	Transcription factor, cholesterol	Therapeutic target	(71, 74, 75, 79)
ChREBP	Transcription factor	Therapeutic target	(82, 83, 85)
ABCA1	Cholesterol	Therapeutic target	(189, 190, 191)
OSBPL7	Cholesterol	Therapeutic target	(168, 199, 200)
Ceramide	Sphingolipid	Therapeutic target, Biomarker	(204, 208, 209, 213, 226)
Sphinganine, Sphingosine	Sphingolipid	Biomarker	(215)
Glucosylceramide	Sphingolipid	Therapeutic target	(228, 229)
Cers6	Sphingolipid	Therapeutic target	(226)
S1P (Sphingosine-1-phosphate)	Sphingolipid	Therapeutic target	(231, 237, 239, 240)
SMPDL3b	Sphingolipid	Therapeutic target	(242, 243)
C1P (Ceramide-1-phosphate)	Sphingolipid	Therapeutic target	(242, 243)
ACSS2	Fatty acid	Therapeutic target	(77, 78)
FASN	Fatty acid	Therapeutic target	(77, 78)
PGC-1α	Transcription factor, Mitochondria	Therapeutic target	(275, 321)
AMPK	Mitochondria	Therapeutic target	(274, 275)
DRP-1	Mitochondria	Therapeutic target	(264)
NDUFS4	Mitochondria	Therapeutic target	(266)
Metrnl	Mitochondria	Therapeutic target	(275)
Parkin	Mitochondria	Therapeutic target	(269, 271)
TNF	Inflammation	Therapeutic target, Biomarker	(328)
IL-1β	Inflammation	Therapeutic target, Biomarker	(328)
IL-6	Inflammation	Therapeutic target, Biomarker	(329)
MCP-1	Inflammation	Therapeutic target, Biomarker	(324, 330, 331)
CRP	Inflammation	Biomarker	(334)
ICAM1	Inflammation	Therapeutic target, Biomarker	(4)
VCAM1	Inflammation	Therapeutic target, Biomarker	(4)
TNFR1/2	Inflammation	Biomarker	(336)
STING	Inflammation	Therapeutic target	(337)
cGAS	Inflammation	Therapeutic target	(338)
NLRP3	Inflammation	Therapeutic target	(339)
IL-18	Inflammation	Biomarker	(339)
STAT3	Inflammation	Biomarker	(340)
NRF2	Inflammation, cell injury	Therapeutic target	(342, 349, 350)
GPX4	Cell injury	Therapeutic target	(345)
RIPK1/3	Cell injury	Therapeutic target	(356, 358)
PAD4	Cell injury	Therapeutic target	(361)
BAX	Cell injury	Therapeutic target	(369)
BCL2	Cell injury	Therapeutic target	(369)
Caspase 11	Cell injury	Therapeutic target	(373)
GSDMD	Cell injury	Therapeutic target	(373)
NLRC5	Cell injury	Therapeutic target	(383)
FDX1	Cell injury	Therapeutic target	(385)
DNMT1/3	Epigenetics	Therapeutic target	(428, 429)
EZH2	Epigenetics	Therapeutic target	(467–469)
LncRNA H19	Epigenetics	Therapeutic target	(480)
LncRNA LINC00968	Epigenetics	Therapeutic target	(481)
LncRNA *Tug 1*	Epigenetics	Therapeutic target	(482)
miR-503	Epigenetics	Therapeutic target	(483)
miR-192	Epigenetics	Therapeutic target	(484)

ABCA1, ATP-binding cassette transporter A1; ACSS2, acyl-CoA synthetase short-chain family 2; AMPK, AMP-activated protein kinase; CEP, C-reactive protein; cGAS, cyclic GMP-AMP synthase; ChREBP, carbohydrate response element-binding protein; DNMT1/3, DNA methyltransferases 1/3; DRP-1, dynamin-related protein 1; ERRα, estrogen-related receptor alpha; ERRγ, estrogen-related receptor gamma; EZH2, enhancer of zeste homolog 2; FASN, fatty acid synthase; GPX4, glutathione peroxidase 4; GSDMD, gasdermin D; ICAM-1, intercellular adhesion molecule-1; IL-1β, interleukin-1 beta; IL-6, interleukin-6; LncRNA H19, long noncoding RNA H19; LXRα/b, liver X receptor alpha/beta; MCP-1, monocyte chemoattractant protein-1; MR, mineralocorticoid receptor; NLRP3, nucleotide-binding oligomerization domain-like receptor family pyrin domain-containing 3; OSBPL7, oxysterol binding protein like 7; PGC-1α, proliferator-activated receptor gamma coactivator-1 alpha; PPARα, Peroxisome proliferator-activated receptor alpha; SMPDL3b, sphingomyelin phosphodiesterase acid-like 3 b; SREBP, sterol regulatory element-binding protein; STAT3, signal transducer and activator of transcription 3; TGR5, Takeda G-protein-coupled receptor 5; TNFR, tumor necrosis factor receptors; VCAM-1, vascular cell adhesion molecule-1; VDR, vitamin D receptor.
